# Using AlphaFold-Multimer to study novel protein-protein interactions of predation essential hypothetical proteins in *Bdellovibrio*

**DOI:** 10.3389/fbinf.2025.1566486

**Published:** 2025-04-14

**Authors:** Ibukun John Abulude, Isabel Cristina Rodríguez Luna, Alejandro Sánchez Varela, Andrew Camilli, Daniel E. Kadouri, Xianwu Guo

**Affiliations:** ^1^ Laboratorio de Biotecnología Genómica, Centro de Biotecnología Genómica, Instituto Politécnico Nacional, Cd Reynosa, Tamaulipas, México; ^2^ Department of Molecular Biology and Microbiology, Tufts University School of Medicine, Boston, MA, United States; ^3^ Department of Oral Biology, Rutgers School of Dental Medicine, Newark, NJ, United States

**Keywords:** *Bdellovibrio*, hypothetical proteins, protein-protein interactions, alphafold-Multimer, attack phase

## Abstract

Bdellovibrio *bacteriovorus* is the most studied member of a group of small motile Gram-negative bacteria called *Bdellovibrio* and Like Organisms (BALOs). *B. bacteriovorus* can prey on Gram-negative bacteria, including multi-drug resistant pathogens, and has been proposed as an alternative to antibiotics. Although the life cycle of *B. bacteriovorus* is well characterized, some molecular aspects of *B. bacteriovorus*-prey interaction are poorly understood. Hypothetical proteins with unestablished functions have been implicated in *B. bacteriovorus* predation by many studies. Our approach to characterize these proteins employing Alphafold has revealed novel interactions among attack phase-hypothetical proteins, which may be involved in less understood mechanisms of the *Bdellovibrio* attack phase. Here, we overlapped attack phase genes from *B. bacteriovorus* transcriptomic data sets and from transposon sequencing data sets to generate a set of proteins that are both expressed at the attack phase and are necessary for predation, which we termed Attack Phase Predation-Essential Proteins (AP-PEP). By applying Markov Cluster Algorithm and AlphaFold-Multimer to analyze the protein network and interaction partners of the AP-PEPs, we predicted high-confidence protein-protein interactions and two structurally similar but unique novel protein complexes formed among proteins of the Bd2209-Bd2212 and Bd2723-Bd2726 operons. Furthermore, we confirmed the interaction between hypothetical proteins Bd0075 and Bd0474 using the Bacteria Adenylate Cyclase Two-Hybrid system. In addition, we confirmed that the C-terminal domain of Bd0075, which contains Tetratricopeptide repeat motifs, participates principally in its interaction with Bd0474. This study revealed previously unknown cooperation among predation essential hypothetical proteins in the attack phase *B. bacteriovorus* and has paved the way for further work to understand molecular mechanisms of BALO predation processes.

## 1 Introduction

*Bdellovibrio bacteriovorus* is a Gram-negative obligate predator that can prey on a wide range of Gram-negative bacteria, including multi-drug resistant pathogens such as clinical isolates of *Acinetobacter baumannii* and *Psuedomonas aeruginosa*, and has been proposed as an alternative to antibiotics (Abulude et al., 2023; Cavallo et al., 2021). The life cycle of *Bdellovibrio bacteriovorus* starts with the Attack Phase (AP) where fast-swimming AP cells locate their prey, attach to, and enter into the prey’s periplasm, forming a spherical bdelloblast (Herencias et al., 2020; Makowski et al., 2019). In the Growth Phase (GP), *Bdellovibrio* consumes the prey’s cytoplasmic content to supply nutrients for multiplication. When nutrients are depleted, the *Bdellovibrio* cells escape to start a new attack cycle (Ajao et al., 2022; Evans et al., 2007).

The life cycle of *B. bacteriovorus* is well characterized, and several genes essential for predation have been identified. However, many molecular aspects of *B. bacteriovorus* predation and growth remain unknown (Dwyer and Volle, 2019; Lai et al., 2023; Medina et al., 2008; Rotema et al., 2015). Arguably, hypothetical proteins may help fill this knowledge gap. This hypothesis becomes more compelling, considering many genes identified as important for predation in *B. bacteriovorus* code for hypothetical proteins whose molecular functions are unknown. For example, in one study, of 16 genes identified as “predation-essential” 7 coded for hypothetical proteins (Tudor et al., 2008). Hypothetical proteins also constituted 72% of 240 “predatosome” genes identified by Lambert and coworkers as specifically upregulated during predation (Lambert et al., 2010). Moreover, hypothetical proteins formed the largest functional category (39.42%) of 104 genes listed as essential for predation during genome-wide transposon sequencing (Tn-seq) characterization of gene function in *B. bacteriovorus* (Duncan et al., 2019). In a recent study, Caulton et al. revealed the MAT superfamily, a group of trimeric fiber proteins with diversified adhesive tips that function as prey recognition moieties (Caulton et al., 2024). Four of the six MAT proteins implicated in prey recognition are annotated as hypothetical proteins in the *B. bacteriovorus* HD100 genome as of this writing (Caulton et al., 2024). The abundance of hypothetical proteins implicated in *B. bacteriovorus* predation calls for further studies.

In this study, we cross-referenced genes from two transcriptomic data sets of *B. bacteriovorus* (Karunker et al., 2013; Lambert et al., 2010) with a Tn-seq data set (Duncan et al., 2019) to create an overlapping set of proteins expressed at the attack phase and necessary for predation. We refer to these as “Attack Phase-Predation-Essential Proteins (AP-PEP)”. Using AlphaFold-Multimer, we predicted protein-protein interactions among AP-PEP proteins. Using the Bacterial Two-Hybrid system, we showed that Bd0075, containing Tetratricopeptide repeat (TPR) domains, interacts with Bd0474, a forkhead-associated (FHA) domain-containing protein. Also, as predicted by Alphafold, we demonstrated that the C-terminal domains of both proteins are responsible for the interaction. Furthermore, we report two structurally similar novel protein complexes formed by the Bd2209-Bd2212 and Bd2723-Bd2726 operons.

## 2 Materials and methods

### 2.1 Strains and culture conditions

*Escherichia coli* strains XL1Blue and BTH101 were grown in Luria Bertani (LB) with 10 μg/mL tetracycline and 100 μg/mL streptomycin, respectively, and were maintained at 37°C shaking. *Bdellovibrio bacteriovorus* 109J was co-cultured with *E. coli* DH5α prey in HEPES buffer at 29°C shaking as described previously (Jurkevitch, 2012).

### 2.2 Clustering of orthologous proteins

Proteins expressed in the attack phase were obtained from the transcriptomic data of Lambert and coworkers (Lambert et al., 2010) and Karunker and coworkers (Karunker et al., 2013). These, together with genes from the Tn-seq data of Duncan and coworkers (Duncan et al., 2019), were mapped for overlaps using OrthoVenn3 (Sun et al., 2023), which clustered orthologous or identical proteins.

### 2.3 Protein sequence annotation, domain characterization and structural modelling

InterPro (Blum et al., 2021) was used to scan the input amino acid sequences for families, conserved domains, and sites by sequence comparison. InterPro and PHOBIUS (Käll et al., 2004) were used to predict the transmembrane topology of proteins and to annotate their amino acid sequences in cytoplasmic, intermembrane, and non-cytoplasmic regions (Blum et al., 2021). NCBI’s Conserved Domain Database (CDD) (Marchler-Bauer et al., 2017) was used to find conserved domains. SWISS-MODEL (Waterhouse et al., 2018) and Foldseek (van Kempen et al., 2023) were used for 3D-homology searches. US-align (Zhang et al., 2022) was used for structural analysis of proteins and protein complexes.

### 2.4 Physicochemical properties of proteins

The ExPASy Protopam server at https://web.expasy.org/cgi-bin/protparam/protparam (Walker et al., 2005) was used to predict physicochemical properties. Molecular weight, theoretical pI, amino acid composition, atomic composition, instability index, aliphatic index, and grand average of hydropathicity (GRAVY), amongst other physicochemical properties, were deduced from the primary protein sequences of the proteins.

### 2.5 Estimation of binding affinities

Putative binding affinities of protein-protein interactions and complexes were estimated using PRODIGY (Vangone and Bonvin, 2017).

### 2.6 Protein-protein interaction network prediction

Protein association networks were predicted using the STRINGS database (von Mering et al., 2003). STRINGS output was visualized using Cytoscape3.9 (Shannon et al., 2003). Protein-protein interactions (PPI) were predicted by AlphaFold2-multimer version 3 *via* the ColabFold server (Evans et al., 2022; Mirdita et al., 2022). AlphaFold-Multimer’s interface predicted template modeling (ipTM) score <0.55 has been shown to indicate random predictions, while 0.55–0.85 performs better than random, with increasing accuracy (Homma et al., 2024; O’reilly et al., 2023; Zhu et al., 2023). The predicted interaction models were viewed and analyzed using ChimeraX version 1.5 (Pettersen et al., 2021).

### 2.7 Bacterial adenylate cyclase two-hybrid test

Protein-protein interaction partners were confirmed experimentally using the Bacterial Adenylate Cylase Two-Hybrid (BACTH) system (Euromedex No. EUK001). Each of the two genes coding for proteins that could interact was inserted into either pUT18C/pUT18 or pKT25/pKTN25 BACTH plasmids. This allows each protein to fuse with BACTH *Bordetella pertussis* adenylate cyclase 18 or 25 subunits, respectively. These plasmid constructions were co-transformed into chemically competent *E. coli* BTH101 and transformants were selected on LB agar plates supplemented with 0.5 mM isopropyl-β-D-thiogalactopyranoside (IPTG), 40 μg/mL 5-bromo-4-chloro-3-indoyl-β-D-galactopyranoside (X-Gal), 50 μg/mL kanamycin and 100 μg/mL ampicillin, incubated for 36 h at 30°C. A co-transformation of *E. coli* BTH101 with pKT25-zip and pUT18C-zip was a positive control in the test.

### 2.8 Construction of specific fragments for protein domains

Primers were designed to amplify nucleotide sequences corresponding to different domains of the Bd0075 and Bd0474 proteins. Engineered proteins that excluded domains were constructed by fusing nucleotide sequences upstream and downstream of the excluded region by Splicing by Extension Overlap (SOE) PCR. The resulting amplicons were cloned directionally into BACTH expression vector pKT25 or pUT18C.

## 3 Results

### 3.1 Consensus predation-essential-hypothetical proteins

**FIGURE 1:**
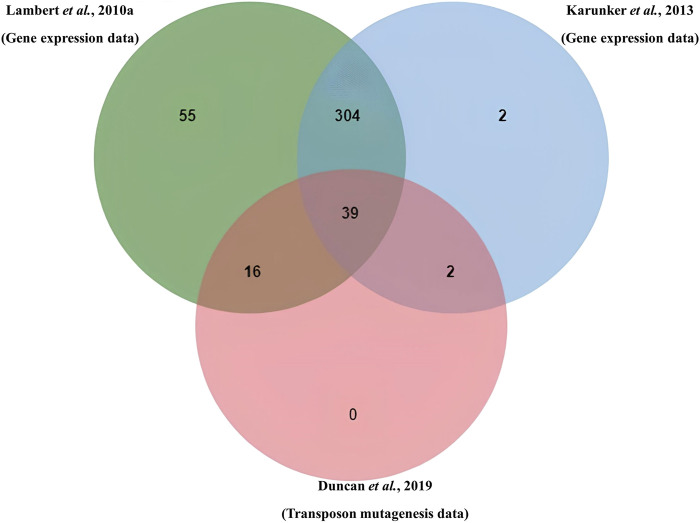
Venn diagram shows overlap and cluster among AP hypothetical proteins from RNA expression data sets (Lambert et al. and Karunker et al.) and hypothetical proteins from transposon data (Duncan et al.).

From the transcriptomics data published by Lambert et al. (2010), genes involved in the attack phase (AP) phase totaled 1,535 after eliminating redundancy caused by duplicates across the categories. Of these, sequences of 1,456 proteins were recoverable from NCBI GenBank, likely due to genome reannotation. Similarly, of the 421 AP genes from the transcriptomics data set of Karunker et al. (2013), 411 protein sequences were recoverable, while 101 of 105 proteins from Tn-seq data set from Duncan et al. (2019) were recovered from NCBI GenBank. Using OrthoVenn to cluster orthologous proteins from the three data sets, a total of 818 proteins were clustered, while 1,064 proteins were unclustered (singletons) (Table 1). As shown in Figure 1, the overlapping region of the three data sets had 39 clusters containing 43 proteins. The 43 proteins, which are expressed in the attack phase and essential for predation (AP-PEP), are listed in Supplementary S4. Proteins from all the input datasets and clusters in all overlaps in the data sets are provided in Supplementary S1.

**TABLE 1:** Summary of clustering of orthologous proteins in the three data sets.

Data sets		Proteins	Number of clusters	Singletons (unclustered)
Transcriptomic Data	Lambert et al. (2010)	1,456	414	961
	Karunker et al. (2013)	411	347	59
Transposon Data	Duncan et al. (2019)	101	57	44

### 3.2 Prediction of protein-protein associations and MCL clustering with STRINGS

Each of the 43 proteins from the overlap among three datasets was used as a query in the STRINGS database to predict functional protein associations. This resulted in 43 “local” interaction networks containing 169 unique proteins after eliminating redundancy caused by duplications. Using the 169 proteins, a “global” protein interaction network was constructed. To facilitate the identification of potential protein complexes or structures within the global network, we applied the Markov Cluster Algorithm (MCL) with an inflation value of 3. This analysis formed 27 distinct clusters, which were derived based on stochastic flow dynamics. Hypothetical proteins were found in interactions with other proteins across different clusters. Proteins in each cluster are given in Supplementary S2.

### 3.3 Prediction of direct protein-protein interaction with AlphaFold-Multimer

**FIGURE 2:**
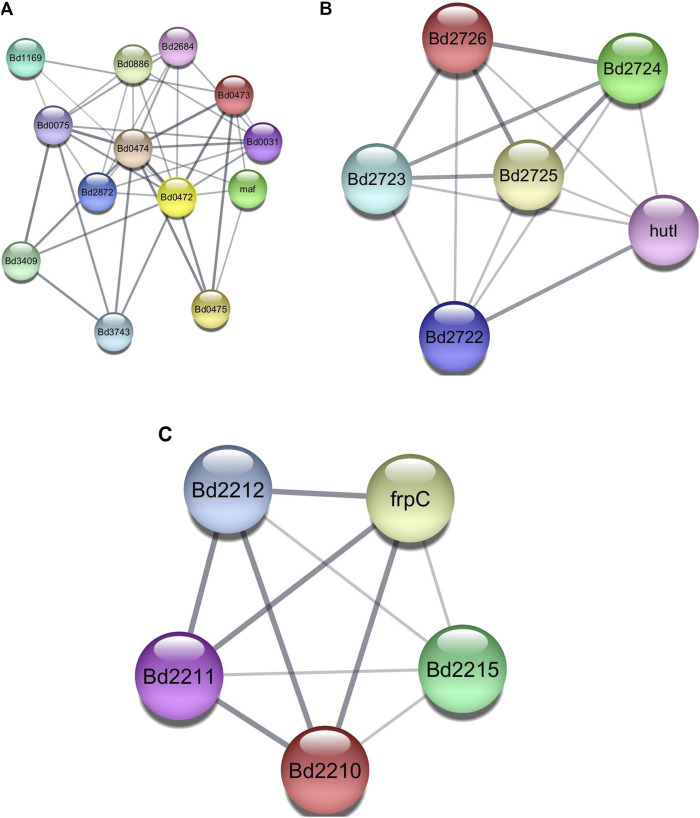
**(A)** CLUSTER 4, containing proteins that include Fork Head Associated (FHA) domains and Tetratricopeptide Repeat (TPR) domains. **(B)** CLUSTER 10, containing proteins from the Bd2723 - Bd2726 operon. **(C)** CLUSTER 12, containing proteins from the Bd2209 - Bd2212 operon.

Employing AlphaFold-Multimer, we explored clusters 4, 10, and 12 (Figure 2) for direct protein-protein interactions. Cluster 4 forms an interesting group as most proteins have one or more Forkhead-associated (FHA) or TPR domains known to play roles in protein-protein interaction. Clusters 10 and 12 had members who formed complete operons. Cluster 12 had four of its five proteins annotated as hypothetical proteins.

All interactions within each cluster were assigned interaction scores by STRINGS based on criteria such as gene neighborhood, gene fusions, co-expression, and gene co-occurrence across genomes. STRING-combined Scores above the cut-off ≥0.7, based on the lowest score of a set of experimentally established protein-protein interactions (positive controls), were considered relevant. STRING-predicted interactions above the cut-off were tested for direct protein-protein interaction in AlphaFold-Multimer.

**FIGURE 3:**
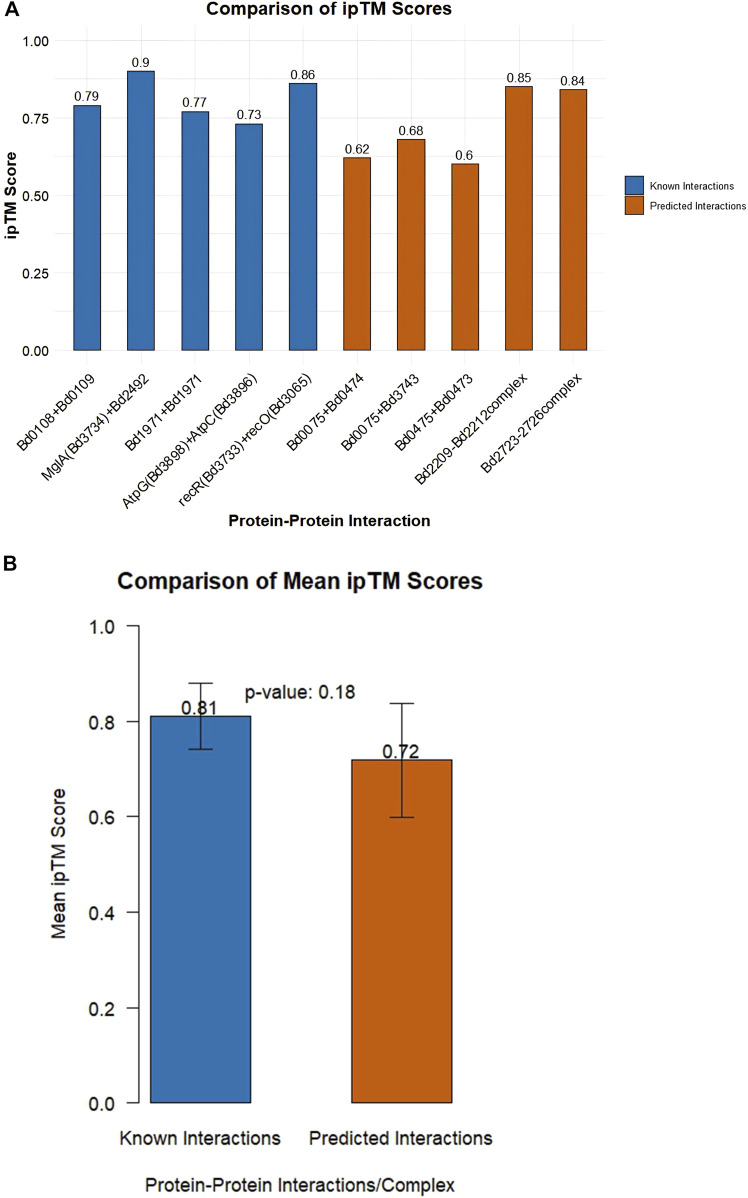
**(A)** Comparison of ipTM scores among known interactions (positive controls) and unknown interactions **(B)** Comparison of mean ipTM scores of known and unknown interactions. A p-value of 0.18 indicates no statistically significant difference between the means as determined by the t-test.

AlphaFold-Multimer ipTM (interface predicted template modeling) scores between 0.6 and 0.8 are confident while iPTM scores >0.8 are highly confident. AlphaFold-Multimer ipTM scores of interactions are given in Supplementary S3. A summary of positive interactions by AlphaFold-Multimer (i.e., ipTM score above 0.6) is shown in Table 2. A comparison between mean ipTM scores between positive controls (known interactions) and predicted interactions yielded a p-value of 0.18. This indicates that there is no statistically significant difference between the means, as determined by the t-test, Figure 3.

**TABLE 2:** Summary of interaction positive by AlphaFold-multimer.

Predicted interaction	STRINGS combined score	Interacting members from the same operon	Predicted AlphaFold multimer interaction/Complex	AlphaFold-multimer pTM and ipTM scores of protein interaction or complex
Bd0075 + Bd0474	0.876	—	Yes (interaction)	pTM = 0.552 ipTM = 0.619
Bd0075 + Bd3743	0.721	—	Yes(interaction)	pTM = 0.66 ipTM = 0.676
Bd0475 + Bd0473	0.823	Operon	Yes(interaction)	pTM = 0.497 ipTM = 0.604
Bd2212 + frpC(Bd2209)	0.773	Operon	Yes (Complex)	pTM = 0.819 ipTM = 0.852
Bd2212 + Bd2210	0.773			
Bd2212 + Bd2211	0.786			
Bd2726 + Bd2725	0.773	Operon	Yes(Complex)	pTM = 0.808 ipTM = 0.837
Bd2726 + Bd2724	0.773			
Bd2726 + Bd2723	0.701			
MglA_Bd3734_ + Bd2492 (Milner et al., 2014)	0.7240	—	Positive control (interaction)	pTM = 0.61 ipTM = 0.898
Bd108 + Bd109 (Prehna et al., 2014)	0.741	Operon	Positive control (interaction)	pTM = 0.804 ipTM = 0.789
Bd 1971 + Bd 1971 (Cadby et al., 2019)	—	—	Positive control (interactiom)	pTM = 0.818 ipTM = 0.772
AtpG_Bd3898_ + AtpC_Bd3896_ (Cingolani and Duncan, 2011)	0.999	Operon	Positive control (interaction)	pTM = 0.79 ipTM = 0.729
RecR_Bd3733_ + RecO_Bd3065_ (Umezu et al., 1993)	0.999	—	Positive control (interaction)	pTM = 0.84 ipTM = 0.859

### 3.4 Complexes from clusters 10 and 12

**FIGURE 4:**
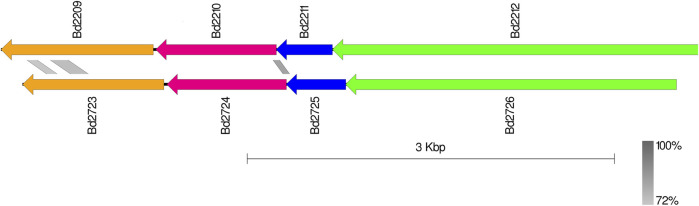
Similar genomic structure of the Bd2209-Bd2212 and Bd2723-Bd2726 operons. Blast identity scale (grey) shows limited identity in the operons (Bd2209/Bd2723 : 39.14%, Bd2210/Bd2724 : 36.56%).

**FIGURE 5:**
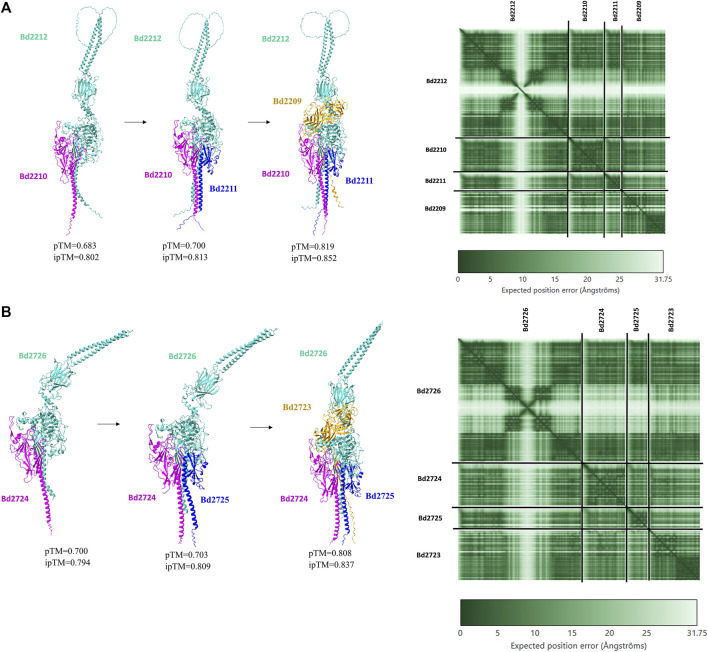
**(5Ai)** AlphaFold-multimer model of Bd2209-Bd2,212 complex **(5Aii)** Predicted Aligned Error (PAE) matrices, showing confidence in structural predictions for Bd2209-Bd2212 complex **(5Bi)** AlphaFold-multimer model of Bd2723-Bd2,726 complex **(5Bii)** Predicted Aligned Error (PAE) matrices, showing confidence in structural predictions for Bd2723-Bd2726 complex.

Bd2723, Bd2724, Bd2725, and Bd2726 in cluster 10 are encoded by genes within an operon, while the genes encoding Bd2209, Bd2210, Bd2211, and Bd2212 in cluster 12 constitute another operon. Each operon encodes for proteins that form predicted protein complexes comprising all four member proteins. Interestingly, the two operons have similar gene arrangement, and corresponding pairs from the two operons have similar sizes (Figure 4). BLAST analysis shows that corresponding gene pairs have low percentage identities. BLAST E-values and percentage identities are shown in Table 3. Simulation of the predicted complexes, in AlphaFold-Multimer by stepwise addition of each protein (in order of largest to smallest) gave highly confident ipTM scores at each step. The largest proteins in the two operons, Bd2212 and Bd2726, respectively, acted like hubs into which other proteins fitted. The predicted structure of each complex is shown in Figure 5. The complexes exhibit a similar overall structure with a TM-score of 0.80356, indicating that they share highly similar folds. However, the RMSD of 4.17 Å suggests that there are differences at the atomic level, particularly in their fine details. The superimposed structure of the complexes is given in Supplementary S5.

**TABLE 3:** Blast E-values and percentage identities of corresponding protein pairs from Bd2209-Bd2,212 and Bd2723-Bd2,726 operons.

NCBI BLAST percentage identity						
Genes	Bd2209 (414 aa)	Bd2210 (328 aa)	Bd2211 (153 aa)	Bd2212 (995 aa)	Blast E-values	Querry Cover (%)
Bd2723 (384 aa)	39.14	—	—	—	1e-93	95
Bd2724 (325 aa)	—	36.56	—	—	3e-67	95
Bd2725 (162 aa)	—	—	41.36	—	7e-48	99
Bd2726 (901 aa)	—	—	—	31.49	4e-146	98

### 3.5 Proteins and interaction from cluster 4

From cluster 4, three interactions Bd0075 + Bd0474, Bd0075+ Bd3473, and B0475 + Bd0473 having ipTM >0.6, were selected for further analysis.

#### 3.5.1 Bd0075 and Bd0474 protein structures and interaction

**FIGURE 6:**
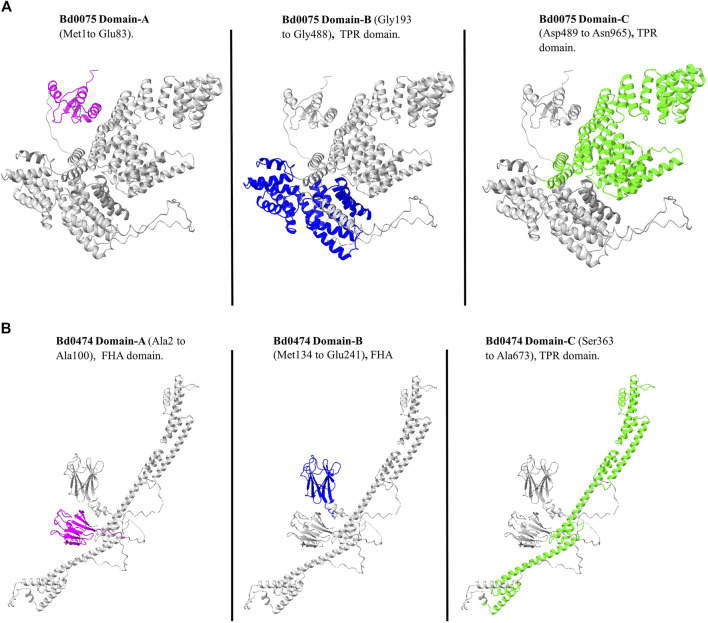
**(A)** The Bd0075 protein. Domain-A (Purple) Domain-B (Blue) and Domain-C (Green). **(B)** The Bd0474 protein. Domain-A (Purple), Domain-B (Blue), and Domain-C (Green).

The AlphaFold model of Bd0075 shows three distinct domains termed A, B, and C. Using InterPro, Domain-A (Met1 to Glu83), which had no annotation, is predicted to be in the cytoplasm. In contrast, Domain-B (Gly193 to Gly488) and Domain-C (Asp489 to Asn965), containing TPR repeats, are predicted to be extracytoplasmic (Figure 6A). The AlphaFold structure of Bd0474 also showed three distinct Domains. Domain-A (from Ala2 to Ala100) and B (from Met134 to Glu241) are FHA domains and predicted to be in the cytoplasm, while Domain-C (from Ser363 to Ala673) containing TPR repeats is extracytoplasmic (Figure 6B).

**FIGURE 7:**
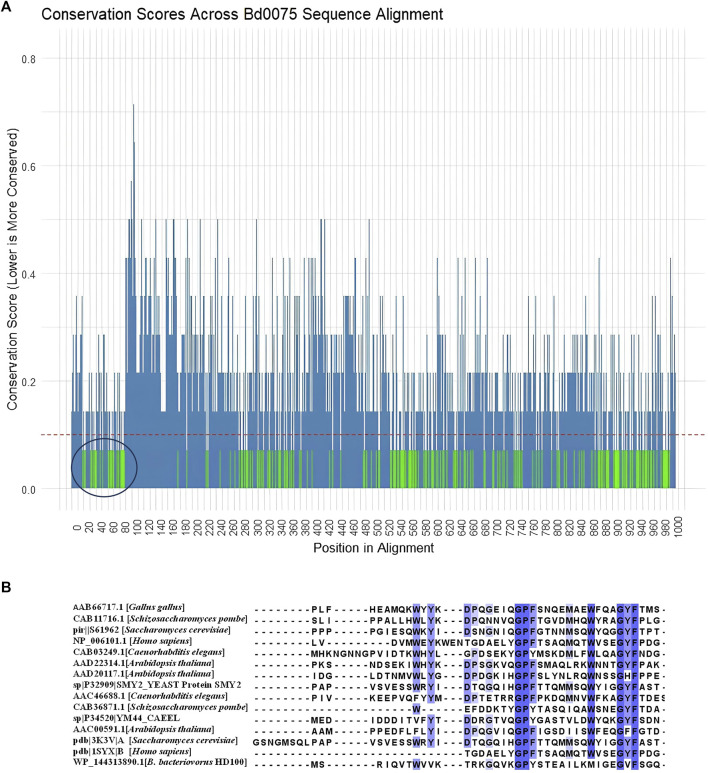
**(A)** A plot showing the conservation scores across the alignment of Bd0075 from 14 *Bdellovibrio* species, with conservation scores plotted on the y-axis and positions in the alignment on the x-axis. Regions highlighted in green represent positions with conservation scores below a threshold of 0.1, indicating that these positions are conserved across species. Conversely, positions with higher conservation scores (above 0.1) are shown in steel blue, indicating lower conservation and greater variability among the sequences. The region circled black shows the conservation of the Domain-A of Bd0075. **(B)** Multiple alignment of protein representatives of the GYF domain from NCBI-CDD with Bd0075 of *Bdellovibrio bacteriovorus* HD109 J.

To check the conservation of residues within each domain in Bd0075, we calculated conservation scores for each aligned position based on a multiple sequence alignment of 14 *Bdellovibrio* sequences (both intraperiplasmic and epibiotic *Bdellovibrio* species) (Supplementary S4). A threshold of 0.1 was established to identify highly conserved positions, indicating that at these sites, 90% or more of the sequences exhibit identical residues. The green-colored regions in the conservation plot represent positions with scores below this threshold and are thus considered statistically significant for conservation (Figure 7A). These conserved positions likely correspond to functionally important areas within the protein structure. As expected, the regions corresponding to the known TPR domains in Bd0075 were conserved. In addition, the N-terminal region corresponding to the Bd0075 Domain-A is also conserved.

We therefore sought the unannotated Domain-A based on structural homology with known protein structures. Using the first 166 amino acids of Bd0075 which corresponds to the cytoplasmic domain region within which the Domain-A is found, a 3D-homology search using SWISS-MODEL shows that the Domain-A shares structural similarities to the glycine-tyrosine-phenylalanine (GYF) domain of the human CD2 cytoplasmic domain binding G protein (CD2BP2) found in the intracellular CD2 binding protein 2 (CD2BP2). This domain has the conserved motif, GP [YF]xxxx [MV]xxWxxx [GN]YF. A multiple sequence alignment of representative GYF-containing proteins from NCBI-CDD, including those from humans, chicken, and yeast, with the GYF-domain of *B. bacteriovorus* 109J Bd0075 is shown in Figure 7B. Despite being from a prokaryotic origin, Bd0075 aligns well with the motif from these eukaryote proteins. In Bd0075, the use of isoleucine at position 8 of the motif is consistent with representative species like *Arabidopsis thaliana* and *Schizosaccaromyces pombe*. However, Bd0075 substitutes Tryptophan for Methionine at position 11 (Figure 7B). In addition, using Foldseek for 3D-homology search, several Foldseek member databases including CATH50 and AFDB-proteome, gave protein hits with portions matching the GYF-domain. Structural matches of Bd0075 Domain-A and portions of the protein hits, with their respective TM and RMSD are given in Supplementary S5.

**FIGURE 8:**
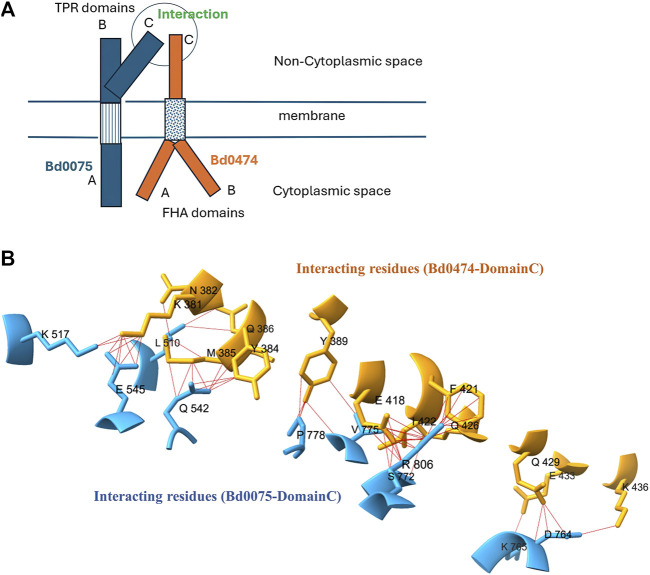
**(A)** Cartoon of a Bd0075-Bd0,474 interaction in the membrane **(B)** Close-up view of 3D structure of domain-domain interactions between Bd0075 and Bd0474.

Using AlphaFold-Multimer, we predicted an interaction between Bd0075 and Bd0474 with an ipTM score of 0.619, interaction model is given in Supplementary S5. Although this score falls in the grey area, an ipTM range 0.6–0.8, where predictions may be correct or wrong (https://www.ebi.ac.uk/training/online/courses/alphafold/inputs-and-outputs/evaluating-alphafolds predicted-structures-using-confidence-scores/confidence-scores-in-alphafold-multimer/), our experimental analysis validated this interaction. Moreover, some known interactions, employed as positive control in this study, had ipTM scores in the 0.6–0.8 range. The Bd0075-Bd0474 interaction is predicted to occur among amino acid residues in the C-Domain of both proteins co-located in the extracytoplasmic space. AlphaFold-Multimer model of the Bd0075-Bd0474 interaction surface showing interacting residues, and a cartoon representation of how they might interact in the membrane is given in Figure 8. Interaction model of the complete domains is given in Supplementary S5.

#### 3.5.2 Bd0075 and Bd3743 interaction

Bd0075 also showed a significant interaction (ipTM = 0.676) with the helix-turn-helix domain-containing protein Bd3743. Bd3743 contains an OmpR/PhoB-type DNA-binding domain found in response regulators at its C-terminal but lacks the phosphoacceptor receiver (REC) domain at its N-terminal, it instead possesses a TPR domain. Interestingly, in AlphaFold-Multimer models, Bd3743 binds to Bd0075 at the same sites where Bd0474 binds to Bd0075. The amino acid residues in Bd0075 involved in binding Bd0474 and Bd3743 are shown in Table 4. Modeling interaction dynamics using the three proteins in AlphaFold-Multimer shows that the Bd0075 TPR domain prefers Bd0474 in the presence of Bd3743 (Supplementary S4).

**TABLE 4:** Bd0075 amino acid residues participating in interactions with Bd0474 or Bd3743.

Bd0075	Bd0474	Bd3743
**LEU510**	**ASN382**	**ALA111**
	**MET385**	
	**GLN386**	
**GLN542**	**TYR384**	**LEU110**
	**MET385**	
**VAL775**	**TYR389**	**PHE154**
	**ILE422**	**VAL157**
**PRO778**	**TYR 389**	**ASN150**
		**VAL157**
**ARG806**	**GLU418**	**GLN156**
	**PHE421**	
	**ILE 422**	
SER772	GLN426	—
ASP764	GLN429	—
	LYS436	
LYS765	GLU433	—
PHE544	—	LYS146
LEU771	—	GLN156
		VAL157
ARG745	—	ALA115
		ASP117
THR799	—	ASN150
ARG809	—	TYR149
LEU810	—	ASN150

Residues participating in Bd0075 - Bd0474 and Bd0075 - Bd3743 interactions are in bold font.

#### 3.5.3 Bd0475 and Bd0473 interaction

The proteins Bd0473 and Bd0475 are encoded in the same operon with Bd0470, Bd0471, Bd0472, and Bd0474. In AlphaFold-Multimer, Bd0473 (containing an FHA domain) interacts with Bd0475 (a hypothetical protein) with an ipTM score of 0.604.

### 3.6 Physicochemical properties of proteins and binding affinity of interactions and complexes

Based on their amino acid sequences, we computed physicochemical properties, including instability index, aliphatic index, and GRAVY, for proteins examined in this study. All the proteins in this study except Bd0473, Bd0474, and Bd0475 are predicted to be stable proteins with an instability index below 40 (Table 5). The binding energies of the protein-protein interactions and complexes computed using PRODIGY are shown in Table 6. The more negative the binding energy (-ΔG kcal mol^-1^), the greater the predicted binding affinity of proteins. Binding energies of Bd0075 -Bd0474 interaction ((ΔG = −19.6 kcal mol^-1^) and Bd0075 - Bd3743 (−8.6 kcal mol^-1^)) were consistent with the preferential binding of Bd0075 to Bd0474 in the presence of Bd3743. Binding energies of the Bd2209-Bd2,212 complex (ΔG = −64.7 kcal mol^-1^) and the Bd2723-Bd2726 complex (ΔG = −50.6 kcal mol^-1^) indicate high affinity among the proteins in these complexes.

**TABLE 5:** Physicochemical properties of examined proteins.

Protein	Number of amino acids	Theoretical pI	Instability index	Aliphatic index	Grand average of hydropathicity (GRAVY)
Bd0075	965	7.04	33.06 (stable)	89.91	−0.275
Bd0474	674	5.81	44.86 (unstable)	77.33	−0.553
Bd3743	367	6.43	37.03 (stable)	89.62	−0.362
Bd2212	995	7.43	26.22 (stable)	79.21	−0.296
Bd2211	153	9.06	26.36 (stable)	86.54	−0.239
Bd2210	328	9.11	34.63 (stable)	96.22	−0.110
Bd2209	414	6.91	17.68 (stable)	72.54	−0.228
Bd2726	901	8.28	35.02 (stable)	81.80	−0.406
Bd2725	162	9.04	21.65 (stable)	81.85	−0.112
Bd2724	325	9.15	31.83 (stable)	82.49	−0.145
Bd2723	384	5.72	28.77 (stable)	69.56	−0.311
Bd0475	228	4,94	41.39 (unstable)	96.18	−0.158
Bd0473	386	9.34	54.22 (unstable)	63.70	−0.610

**TABLE 6:** Binding affinities (ΔG), dissociation constant (K_d_), Interfacial contacts (ICs), and percentages Non Interacting Surface (NIS) of examined interactions.

Protein-protein interaction or complex	ΔG (kcal mol^-1^)	K_d_(M) at 30°C	ICs charged-charged	ICs charged-polar	ICs charged-apolar	ICs polar-polar	ICs polar-apolar	ICs apolar-apolar	NIS charged	NIS apolar
Bd0075 +Bd0474	−19.6	6.7e-15	27	21	52	8	42	44	29.91	42.19
Bd0075 +Bd3743	−8.6	4.8e-07	5	8	17	2	12	22	31.27	40.23
Bd0475 +Bd0473	−12.7	6.7e-10	14	16	28	4	22	29	25.54	42.97
Bd2209-Bd2,212 complex	−64.7	2e-47	44	97	137	32	212	232	27.76	37.75
Bd2723-Bd2,726 complex	−50.6	3.2e-37	45	73	138	24	145	239	28.37	41.06

### 3.7 BACTH assay and confirmation of Bd0075 - Bd0474 interaction

We validated one of the predicted interactions, Bd0075 - Bd0474, using a Bacterial Adenylate cyclase two-hybrid assay (BACTH). The positive control was set up as an interaction between the pKT25-Zip and pUT18C-Zip plasmids, which were co-transformed into *E. coli* BTH101 cells and plated on LB plates with 0.5 mM IPTG, 40 μg/mL X-Gal, 50 μg/mL kanamycin and 100 μg/mL ampicillin. The emergence of blue colonies from the plate indicated an interaction. The negative control, between the empty plasmids pKT25 and pUT18C, gave rise to white colonies indicating no interaction. Co-transformation of pKT25:Bd0075 and pUT18C:Bd0474 into BTH101 yielded blue colonies on assay plates, confirming interaction between the proteins.

**FIGURE 9:**
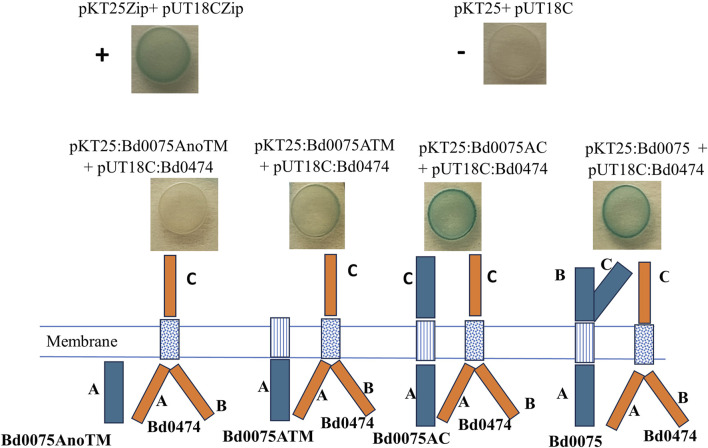
Experimental confirmation of protein-protein interaction by BACTH system. From right to left: Bd0075 interacts with Bd0474, Bd0075 Domain-C participates in the interaction, Transmembrane of Bd0075 may participate in the interaction, and Bd0075 Domain-A without transmembrane has no effect on the interaction.

Furthermore, Bd0075 Domain-A alone (Bd0075AnoTM), Bd0075 Domain-A with transmembrane helix (Bd0075 ATM), and Bd0075 with only Domains A and C (Bd0075 A C) were used in BACTH assay with intact Bd0474. Bd0075AnoTM shows no interaction with Bd0474 as indicated by white colonies from the plate. The Bd0075ATM-Bd0474 gave colonies with slightly blue coloration, suggesting a role for the transmembrane helix in the interaction. The Bd0075AC-Bd0474 interaction yielded blue colored colonies comparable with those from Bd0075-Bd0474 interaction showing that the Domain-C is important for the interaction (Figure 9).

Experiments with different domains of Bd0075 show that using Domain-A alone does not cause the Bd0075-Bd0474 interaction. The inclusion of the Transmembrane helix (TMH) with the Domain-A gave a slight blue color. This could mean that the TMH may contribute to the observed interaction. The interaction tests with engineered Bd0075, containing Domain-C, restored the Bd0075-Bd0474 interaction, showing that, as predicted, the Domain-C participates in the interaction (Figure 9).

### 3.8 Discussion

As *B. bacteriovorus* continues gaining attention as a means of controlling Gram-negative pathogen populations, many molecular aspects of *Bdellovibrio* predation remain unclear, partly due to many hypothetical and uncharacterized proteins with unknown functions within its genome. To assist in narrowing down hypothetical proteins for experimental exploration, we employed AlphaFold-Multimer, an artificial intelligence (AI)-based model developed by DeepMind, which has emerged as a groundbreaking tool for research in macromolecular interactions, significantly advancing the prediction of protein structure and multiprotein complex structures. Using available gene expression and genome-wide transposon sequencing data, we generated a set of 43 Predation Essential Attack Phase Proteins, from which a global protein interaction network involving 169 proteins was constructed. By clustering this network using MCL clustering, we obtained 23 clusters and further investigated interactions from 3 of the clusters.

Only interactions with AlphaFold-Multimer ipTM score above the 0.6 cutoff were considered in this study. IpTM <0.55 has been shown to indicate random predictions, while 0.55–0.85 performs better than random, with increasing accuracy (Homma et al., 2024; O’reilly et al., 2023). We acknowledge that AlphaFold-Multimer has limitations, particularly in its propensity to produce false negative predictions. However, this limitation had minimal impact on our study, as we leveraged AlphaFold-Multimer’s ability to stringently identify positives while maintaining a low false positive rate of approximately 1%, as reported in previous studies (Homma et al., 2024; Johansson-Åkhe and Wallner, 2022; Omidi et al., 2024). This indicates that while some true positives may have been missed, only the most reliable interactions were selected from the vast network for experimental follow-up.

Bd0075 and Bd0474 proteins were predicted to interact with an ipTM score of 0.619. These proteins have been previously implicated alongside other proteins as essential for *B. bacteriovorus* predation by transposon mutagenesis studies (Duncan et al., 2019). Not only are Bd0075 and Bd0474 expressed during the attack phase of predation, but we find, using the MicrobesOnline webserver (https://microbesonline.org/) (Dehal et al., 2009), that their overlaid expression profiles also showed a very strong positive correlation, with a Pearson correlation coefficient of 0.99 suggesting that the proteins are co-expressed and can be simultaneously available for functional interaction in the cell. Analysis of physicochemical properties shows that Bd0474, with an instability index of 44.86, is an unstable protein. Hence Bd0474 might benefit functionally from interacting with a more stable Bd0075 (instability index of 33.06). Our AlphaFold-multimer model of the Bd0075-Bd0474 interaction shows that residues from the extra cytoplasmic Domain-C of Bd0075 and Bd0474 participate in the interaction. Bacterial Adenylate cyclase Two-Hybrid (BACTH) experiments confirmed the Bd0075-Bd0,474 interaction. Furthermore, experiments with interaction using different domains, showed that the Domain-C of Bd0075 is involved in the Bd0075-Bd00474 interaction and that the transmembrane helixes of the proteins may contribute to the interaction and stabilization of the complex. The role of transmembrane helixes in the stabilization of protein-protein interaction and complex in the membrane has been documented (Moore et al., 2008).

In addition, Bd0075 can form bonds with Bd3743 at the same binding site in its Domain-C where Bd0474 binds. Since Bd0474 and Bd3743 seem to “compete” for the same site, we modeled interaction dynamics using the three proteins in AlphaFold-Multimer. Our results showed that TPR site of Bd0075 shows a preference for Bd0474. This was corroborated by results from the calculated binding affinities (ΔG). ΔG of Bd0075-Bd0,474 (−19.6 kcal mol^-1^) is 2.3 times lower than that of Bd0074-Bd3743 (−8.6 kcal mol^-1^). Interestingly, a previous report showed that Bd0474 is downregulated in the growth phase but not in the attack phase. Hence it is attack phase-specific, while Bd3743 is upregulated in both phases (Lambert et al., 2010). The higher affinity of Bd0075 for Bd0474 may allow this preferential binding in the presence of Bd3743 at the attack phase events. Later in the growth phase stage, when Bd0474 cellular level is depleted, Bd3743 could bind to Bd0075.

Bd0075 shows three distinct Domains: A, B, and C. The Domain-B and Domain-C located in the extra-cytoplasmic region contain TPR domains. TPR domains are known to participate in protein-protein interactions and facilitation of protein complexes (Cerveny et al., 2013) and have been identified in proteins playing various roles in vital cell processes, including cell-cycle regulation, transcription, chaperones, and cell signaling (Blatch and Lä Ssle, 1999).

We found that the Domain-A of Bd0075, a small-sized independent domain in the cytoplasmic region of the protein, resembles the GYF domain of the human CD2 cytoplasmic domain binding G protein (CD2BP2). The GYF domain has the conserved motif, GP [YF]xxxx [MV]xxWxxx [GN]YF, and can bind sites containing two tandem PPPGHR segments within the cytoplasmic region of CD2. The existence of the eukaryote-associated GYF domain in *B. bacteriovorus*, could represent the possibility of a eukaryote-like domain being used by *B. bacteriovorus*. Recently, histones, which were generally thought of as being associated exclusively with eukaryotes, have been reported as major chromatin components in *B. bacteriovorus* (Hocher et al., 2023).

Furthermore, we identified two novel complexes in clusters 10 and 12 of our MCL clustered global network. Each of the complexes is constituted by a group of proteins belonging to the same operon. The first complex (Bd2212 complex) is formed by the operon consisting of proteins Bd2209, Bd2210, Bd2211, and Bd2212, while the second complex (Bd2726 complex) is formed by the operon consisting of Bd2723, Bd2724, Bd2725, and Bb2726. These two operons are expressed in the attack phase of predation and comprise mostly unexplored hypothetical proteins. Whereas all proteins in the Bd2212 complex are annotated as hypothetical/unknown proteins, the Bd2726 complex comprises hypothetical/unknown proteins except for Bd2724 (NCBI-Tfp) pilus assembly protein FimT/FimU) and Bd2725 (NCBI- type II secretion system protein). Interestingly, the hypothetical proteins Bd2210, Bd2211, Bd2212, and Bd2723 have been linked with phenotypes with loss of predation in a transposon mutagenesis study (Duncan et al., 2019).

The Bd2209-Bd2212 and Bd2723-2726 operons have a similar arrangement of genes and form structurally similar complexes (TM-score = 0.80356). However, the corresponding genes from both operons have low BLAST percentage identities, suggesting that these gene clusters have undergone significant evolutionary divergence or were obtained from different origins. Additionally, the RMSD value of 4.17 Å of the matched complexes indicates differences at the atomic level. This divergence in gene sequences could reflect functional specialization or adaptation to different environmental conditions or cellular processes. Studying these types of operons could offer valuable insights into the mechanisms of gene duplication, evolution, and functional divergence in *B. bacteriovorus* genomes.

In AlphaFold-multimer models, Bd2212 interacted with other protein members in its operon, forming a complex with a high ipTM score of 0.852, comparable to the most stringent cutoff, iPTM = 0.85, used in a study by O’Reilly and coworkers in studying protein complexes (O’reilly et al., 2023). Likewise, Bd2723 interacted with other members of the Bd2723-2,726 operon to form a similar complex with a high ipTM score of 0.837. The binding affinities (ΔG) of the Bd2212 complex and Bd2723 complex were −64.7 kcal mol^-1^ and -50.6 kcal mol^-1^, indicating a strong affinity among the members for complex formation.

## 4 Conclusion

Using two transcriptomics data and genome-wide transposon sequencing data, this study provides a robust functional interaction network comprising 169 proteins relevant to the *B. bacteriovorus* attack phase. This network was clustered into 23 clusters. With the exploration of 3 clusters, our approach found novel protein-protein interactions and complexes and validated the Bd0075-Bd0,474 interaction. Furthermore, we show that as predicted by AlphaFold-Multimer, the C-Domain of Bd0075 is involved in the interaction. This demonstrates the prospects of this approach for further significant discoveries among the remaining clusters. This study is the first to report the protein complexes involving two operons (Bd2209 to Bd2212 and Bd2723 to Bd2726), similar in genomic structure in *B. bacteriovorus*, which are both expressed in the attack phase. Future work will focus on the experimental exploration of these novel interactions and complexes. Taken together, our approach has not only discovered functional novel interactions and complexes but has also provided templates and resources for further discovery and exploration of these protein interactions with an implication for understanding the underlying molecular mechanisms of *B. bacteriovorus* predation.

## Data Availability

The original contributions presented in the study are included in the article/Supplementary Material, further inquiries can be directed to the corresponding authors.
