# Supplementary material for: Using AlphaFold-Multimer to study novel protein-protein interactions of predation essential hypothetical proteins in Bdellovibrio
Source: Front Bioinform. 2025 Apr 14;5:1566486. doi: 10.3389/fbinf.2025.1566486 (PMC12034629; doi:10.3389/fbinf.2025.1566486)
Supplement: Supplementary file 1 [file DataSheet2.pdf]

Supplementary 4
Table. Consensus Predation-essential hypothetical proteins

	Protein ID	Old Locus Tag	Product Name (NCBI)
1	WP_011162725.1	Bd0110	type II secretion system F family protein
2	WP_011162726.1	Bd0111	ATPase, T2SS/T4P/T4SS family
3	WP_011162727.1	Bd0112	BON domain-containing protein
4	WP_011162728.1	Bd0113	Flp pilus assembly protein CpaB
5	WP_011162729.1	Bd0114	hypothetical protein
6	WP_011162730.1	Bd0115	hypothetical protein
7	WP_011162731.1	Bd0117	hypothetical protein
8	WP_011162732.1	Bd0118	hypothetical protein
9	WP_011162733.1	Bd0119	Flp1 family type IVb pilin
10	WP_157865749.1	Bd0150	hypothetical protein
11	WP_157865623.1	Bd0161	hypothetical protein
12	WP_231839255.1	Bd0470	type II secretion system F family protein
13	WP_011163054.1	Bd0471	DUF192 domain-containing protein
14	WP_011163055.1	Bd0472	FHA domain-containing protein
15	WP_011163056.1	Bd0473	FHA domain-containing protein
16	WP_011163057.1	Bd0474	FHA domain-containing protein
17	WP_011163058.1	Bd0475	hypothetical protein
18	WP_011163298.1	Bd0739	hypothetical protein
19	WP_011163343.1	Bd0791	cytochrome P450
20	WP_226988005.1	Bd0792	cupredoxin domain-containing protein
21	WP_011163345.1	Bd0793	CpaF family protein
22	WP_011163382.1	Bd0832	tetratricopeptide repeat protein
23	WP_011163387.1	Bd0838	biopolymer transporter ExbD
24	WP_011163418.1	Bd0871	hypothetical protein
25	WP_011163557.1	Bd1023	ATP-binding cassette domain-containing protein
26	WP_011163788.1	Bd1290	prepilin-type N-terminal cleavage/methylation domain-containing protein
27	WP_038448782.1	Bd1434	GGDEF domain-containing protein
28	WP_011163964.1	Bd1478	adventurous gliding motility protein U
29	WP_011163965.1	Bd1479	biopolymer transporter ExbD
30	WP_011164234.1	Bd1769	ATP-binding cassette domain-containing protein
31	WP_011164332.1	Bd1877	ABC transporter ATP-binding protein
32	WP_011164398.1	Bd1946	outer membrane beta-barrel protein
33	WP_011164433.1	Bd1981	hypothetical protein
34	WP_041583553.1	Bd2209	EF-hand domain-containing protein
35	WP_011164646.1	Bd2210	hypothetical protein
36	WP_011164647.1	Bd2211	hypothetical protein
37	WP_011164648.1	Bd2212	hypothetical protein
38	WP_041583564.1	Bd2325	HD-GYP domain-containing protein
39	WP_011165116.1	Bd2723	EF-hand domain-containing protein
40	WP_011165510.1	Bd3148	protein kinase

41	WP_011165652.1	Bd3304	hypothetical protein
42	WP_041583662.1	Bd3772	hypothetical protein
43	WP_231839223.1	Bd3839	hypothetical protein

Bd0075 binds Bd0474 in the Prescence of Bd3743

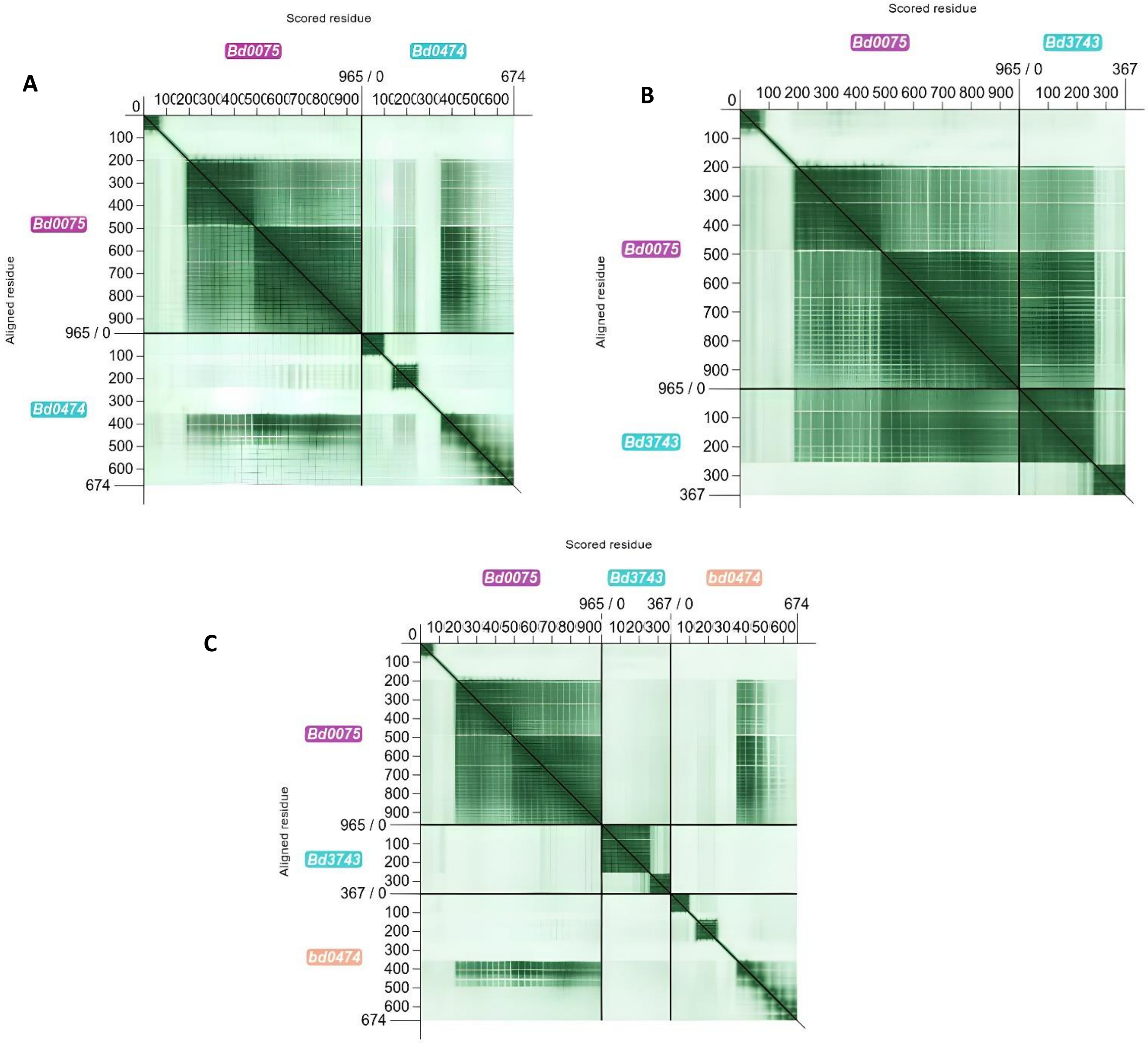

Figure. Predicted Aligned Error plots showing A) Bd0075-Bd0474 interaction, Bd0075 Fold-C interacts with the Bd0474 Fold-C. B) Bd0075-Bd3743 interaction, Bd0075 Fold-C interacts with Fold-A of Bd03743. C) The Fold-C of Bd0075 selectively interacts with Bd0474 in the presence of Bd3743.

Bd0075 sequences from 14 Bdellovibrio sp. aligned to calculate conservation scores.

>B.b_HD100

MSRIQVTWVVKTRKGQVKGPYSTEAILKMIGEGVFSGQEMISKLPDGQWTLISK
EPAFYDKLLEALEGVVDVDPKKAQKMEAETVIVQAPKAPRNNTNTGSTPSPESFANVQPQKPLLQQIDVL
DTPAYVPPAQKIASVSSGDSKSDSVIELSNIKNMEKGEIAKSLKLPALILAVVIVLGVWLLWDTGPADGS
KIHLLAPGKSTATLSDQQIKEKLNEALFAMEQDTFESYVAAQNKLVSIVEGAPMNIEVRALLCVVYFELW
PHAVQDAQDIKTIAGVTQATRALNVISPFGQVCEAVKLMTAGRYKEAKGTIEATLEGSEPFSLLPVLYDF
KAELLAGEKDYVNAVPYYEKAAQLWEKWLHPQVSLAEALYHQGDFTTAAGILRNVLTKNPKHKEAKVFAG
IVEYNGFKKSDTAYAFLNSGLESKGRIPSLIEAEGYGALAEIFVLRGEKKKALEVAQKAFGLNPNNGELR
QLVIRLGGTDKVKGDKGRNNELLYLGDQYVRQGDFLAAQAEFKAAFEADPKNGTAAMKAAKALWQLNQSF
EAIEWLNKAIKAEPKLVSAYVLQADYMSQRFDFIGALQILTNAMRIAPNNYEVLRGLAQLEFRKNNMPGT
VNYALRAAKAYDGDIDTYILLAKANGLLARSIMPINKKEIERKDNASKDAIRYATKAVEIDATNPDAQIT
YAKMLAQTNGVDSGITYLSELIKRFSYTLDYRIALAEVYKSEDRYSQAKDIYEKVTEADPRNKKAWLGLG
ESEKALGLNDKALKAFLSAAVLDPTDGEALFQAGKLYLETSRFEEAIQQFKRVQRLNANYPRTHYYIGKA
AFASGDFATALEASKSEKKLNPNVADSYILAAEVFTARRQYAECAAEYSTAMKLRPQGADIYVKSAQCYR
QSGSLDVAEDMLALASARESGYAEIYREQGAIYEMKGDTRSAAQAYNKYLGLSPNALDRAEIEAKIMRLG
N

>B.b_109J

MSRIQVTWVVKTRKGQVKGPYSTEAILKMIGEGVFSGQEMISKLPDGQWTLISKEPAFYDKLLEALEGVV
DVDPKKAQKMEAETVIVQAPKAPRNNTNTGSTPSPESFANVQPQKPLLQQIDVLDTPAYVPPAQKIASVS
SGDSKSDSVIELSNIKNMEKGEIAKSLKLPALILAVVIVLGVWLLWDTGPADGSKIHLLAPGKSTATLSD
QQIKEKLNEALFAMEQDTFESYVAAQNKLVSIVEGAPMNIEVRALLCVVYFELWPHAVQDAQDIKTIAGV
TQATRALNVISPFGQVCEAVKLMTAGRYKEAKGTIEATLEGSEPFSLLPVLYDFKAELLAGEKDYVNAVP
YYEKAAQLWEKWLHPQVSLAEALYHQGDFTTAAGILRNVLTKNPKHKEAKVFAGIVEYNGFKKSDTAYAF
LNSGLESKGRIPSLIEAEGYGALAEIFVLRGEKKKALEVAQKAFGLNPNNGELRQLVIRLGGTDKVKGDK
GRNNELLYLGDQYVRQGDFLAAQAEFKAAFEADPKNGTAAMKAAKALWQLNQSFEAIEWLNKAIKAEPKL
VSAYVLQADYMSQRFDFIGALQILTNAMRIAPNNYEVLRGLAQLEFRKNNMPGTVNYALRAAKAYDGDID
TYILLAKANGLLARSIMPINKKEIERKDNASKDAIRYATKAVEIDATNPDAQITYAKMLAQTNGVDSGIT
YLSELIKRFSYTLDYRIALAEVYKSEDRYSQAKDIYEKVTEADPRNKKAWLGLGESEKALGLNDKALKAF
LSAAVLDPTDGEALFQAGKLYLETSRFEEAIQQFKRVQRLNANYPRTHYYIGKAAFASGDFATALEASKS
EKKLNPNVADSYILAAEVFTARRQYAECAAEYSTAMKLRPQGADIYVKSAQCYRQSGSLDVAEDMLALAS
ARESGYAEIYREQGAIYEMKGDTRSAAQAYNKYLGLSPNALDRAEIEAKIMRLGN

>B.b_SSB

MSRIQVTWVVKTRKGQVKGPYSTEAILKMIGEGVFSGQEMISKLPDGQWTLISK

EPAFYDKLLEALEGVVDVDPKKAQKMEAETVIVQAPKAPRNNTNTGSTPSPESFANVQPQKPLLQQIDVL

DTPAYVPPAQKIASVSSGESKSDSVIELSNIKNMEKGEIAKSLKIPALILMVVIVLGVFLLWDSGPADGS

KIHLLAPGKSTATLSDQQIKEKLNEALFAMEQDTFESYVAAQNKLVSIVEGAPMNIEVRALLCVVYYELW

PFAVQDAQDIKTIAGITQATRALNVISPFGQVCESVKLMTAGRYKEAKGTIEATLEGSEPFSLLPVLYDF

KAELLAGEKDYVNAVPYYEKAAQLWEKWLHPQVALAQALYHQGDFTSAAGILRNVLTKNPKHKEAKVFAG

IVEYHGFKKSDTAYAFLNSGLESKGRIPSLIEAEGYGALAEIFVLRGEKKKALEVAQKAFGLNPNNGELR

QLVIRLGGTDKVKGEKGRNNELLYLGDQYVRQGDFLAAQAEFKAAFEADPKNGTAAMKAAKALWQLNQSF

EAIEWLNKAIKAEPKLVSAYVLQADYMSQRFDFVGALQILTNAMRIAPNNYEVLRGLAQLEFRKNNMPGT

VNYALRAAKAYDGDIDTYILLAKANGLLARSIMPINKKEIERKENASKDAIRYATKAVEIDATNPEAQIT

YAKMLAQTNGVDSGITYLNELIKRFSYTLDYRIALAEVYKSEDRYSQAKDIYEKVAEADPRNKKAWLGLG

ESEKALGLNDKALKAFLSAAVLDPTDGEALFQAGKLYLETSRFEEAIQQFKRVQRLNANYPRTHYYIGKA

AFASGDFATALEASKSEKKLNPNVADSYILAAEVFTARRQYAECAAEYSTAMKLRPQGADIYVKSAQCYR

QSGSLDVAEDMLALASARESGYAEIYREQGAIYEMKGDTRSAAQAYNKYLGLSPNALDRAEIEAKIMRLG

N

>B.b_Tiberius

MSRIQVTWVVKTRKGQVKGPYSTEAILKMIGEGVFSGQEMISKLPDGQWTLISKEPAFYDKLLEALEGVV

DVDPKKAQKMEAETVIVQAPKTPRNNTNPHSAPSADSFANIQPQKPLLQQIDVLDTPAYVPPAQKIASVS

SGDSKSDSVIELSNIKNMEKGEIAKSLKIPALILMVVIVLGVFLLWDTGPADGSKIHLLAPGKSTATLSD

QQIKEKLNEALFAMEQDTFESYVAAQNKLVSIVEGAPMNIEVRALLCVVYFELWPFAVQDAQDIKTIAGI

TQATRALNVISPFGQVCESVKLMTAGRYKEAKGTIEATLEGSEPFSLLPVLYDFKAELLAGEKDFVNAVP

YYEKAAQLWEKWLHPQVALAEALYHQGDFTSAAGILRNVLAKNPKHKEAKVFAGIVEYNGFKKSDTAYAF

LNSALESKGRIPSLIEAEGFGALAEIFVLRGEKKKALEVAQRAFGLNPNNGELRQLVIRLGGTDKVKGEK

GRNNELLYLGDQYVRQGDFLAAQAEFKAAFEADPKNGTAAMKAAKALWQLNQSFEAIEWLNKAIKAEPKL

VSAYVLQADYMSQRFDFIGALQILTNAMRIAPNNYEVLRGLAQLEFRKNNMPGTVNYALRAAKAYDGDID

TYILLAKANGLLARSIMPINKKEIERKENASKDAIRYATKAVEIDATNPEAQITYAKMLAQTNGVDSGIT

YLNELIKRFSYTLDYRIALAEVYKSEDRYSQAKDIYEKVAEADPRNKKAWLGLGESEKALGLNDKALKAF

LSAAVLDPTDGEALFQAGKLYLETSRFEEAIQQFKRVQRLNANYPRTHYYVGKAAFASGDFATALEASKS

EKKLNPNVADSYILAAEVFTARRQYAECAAEYSTAMKLRPQGADIYVKSAQCYRQSGSLDVAEDMLALAS

ARESGYAEIYREQGAIYEMKGDTRSAAQAYNKYLGLSPNALDRAEIEAKIMRLGN

>B.b_Kdesi

MSRIQVTWVVKTRKGQVKGPYSTEAILRMIGEGVFSGQEMISRLPDGQWTLISKEPAFYDKLLEALEGIV

DVDPKKAQKMEAETVIIQAPKPSKPKTETNTENVFAPPANQKPLLQQIDVLDTPAYIPPAAKIASVPSGG
DKDSVIELSNVKNMEKGELQKSLKVPALILAVVIVLGVWLLWDNGPADGSKIHLLAPGKAGATLSDQAVK
EKLNEALFAMEQDTFESYASAQNKLVSIIEGAPMNIEVRALLCVVYYELWPYAVQDAQDIRTIASVTQAT
RALNVVSPFGQVCEAVKLLTAGRYKEAKGAIEATLESSEPFSLLPVLYSFKAELLAGEKDYLNAVPYFEK
ATQLWEKWLDPQVALAEVLYQQGDFTSSATLLRNVLAKNPKHKEAKVAAGLVEYKGFKKSDTAYSLLNSA
LESKGRIPSLIEAEGYGALAEIFVLRGEKKKALDIAQKAFALNPNNGELRQLVIRLGGSDKIKGEKGRNN
ELLYLGDQYVRQGDFLAAQAEFKAAFEADPKNGTAAMKAAKALWQLNQSFEAIEWLNKSIKAEPKLVSAY
VLQADYMSQRFDFVGALQILTNAMRIAPNNYEVLRGLAQLEFRKNNMVGTINYALRAAKAYDGDIETFIL
LSKANGLLARSIVPINKKEIERKENAAKDAIRYATKAVEIDATNPDAQITYAKMLAQTNGVDSGITYLNE
LIKRFSYTMDYRIALAEVYKSEDRYSQAKEIYERAAEADPRSKKAWLGLGESEKALGLNDRALKAFLSAA
VLDPTDGEALFQAGKLYLETSRFEEAIQQFKRVQRLNPNYPRTHYYVGKAAFASGDFATALEASKAEKKL
NPNVADSYVLAAEVYTARRQYAECAAEYSTAMKLRPQGADIYVKSAQCYRQSGSLDVAEDMLALASARES
GYAEIYREQGAIYEMKGDTRSAAQAYNKYLGLSPNALDRAEIEAKMMRLGN

>B.Reynosensis
MSRIQVTWVVKTRTGQVKGPYSTEAILRMIGEGVFSGQEMISKLPDGQWTLISKEAAFYDKLLEALEGIV
EVDPKKAQKMEAETVIVHPPPQQKPKDSLPSLESFANIKIEKSKQKVDVYDVPQNVQPASIPIANVKSES
VIDLSNIKNMEKGELFKNLRLPAFVLVIVILAAVFLLWEQGPSDGTKIHLLAPKKSTSTMSEEQVKVKLN
EAIFSIEQDTFESYLEAQNKLVTIIEGSGMNLEVRGLLCVVYKELWPFAVQDAQDIKTIVTMTQSTRALN
VISPFGQVCETVKLMTSGRFKEAKGVIEATLESNEPFSLLPVLYDFKAELLRGEHDYTNAIPYYEKASQL
WDKWLNPLVSLAAVSYETGDFNTASSILKNVLAKNPRHREAKILAGIIDYRGFKKSDTAYSYLSTALEAK
GRVAALTLADGYSCLAEIYLLRGEKGKALEAAQSGFDLNPNNSELRQLVIRLGGNDKVKGEKGKNNELLY
LGDQYIRQGDYLAAQAEFKAAFEVDPKNGTAAMKAAKALWQLNQSFEAIEWLNKAIKAEPKLVSAYVLQA
DYMSQRFDFIGALTILTNATRIAPNNYEVLRGLSMLEFRKNNMPGAINYALRSVKAYDGDIEAFILLSKA
NGILARSIMPINKKEIERKENAAKDAIRYATKAVEIDATNPEAQITYAKMLAQTNGVDSGINYLNELIKR
FSYTLDYRIALAEIYKSEDRFTQSKEIYEKVVESDPKNKKAWLGLGESEKALGLNDKALKAFLNAAILDP
TDGEALFQAGKLYLETSRFDEAIQQFKRVQRLNPKYPRTHYYIGKAAFSSGDFTTALEASKMEKKLNPNV
ADSYILAAEVYTARKQYAECAAEYSQAMKLRPQGAEIYVKSATCYRQSGSMDVAEDMLALASARESGYAE
IYREQGAIYELRGDNRSAATAYNKYLGLSPNALDRAEIEMKILRLGGN

>Bd.sp22V
MSRIQVTWVVKTRNGQVKGPYSTEAILRMIGEGVFSGQEMISKLPDGQWSLISKEPAFYDKLLEALEGVV
DVDPKKAQKMEAETVIIQPPRAQKQNPPPPQAPNPESFANLRVEKPKQKVDVYDTPAYVPPAKVPFQSVT
SNDKKSDSVIELSNLKNMEKGEIAKSLKMPAIILAVAIVIGVFLLWDSGPADGSKIHLLAPGKSGATLND
QQVKEKLNEALFAMEQDTFESYIQAQNKLVSIVEGAPMNIEVRALLCVVYKELWPFAVQDAQDVKTIATA

TQQTRALNVISPFGQVCEAVKLMTTGRYKEAKGVVETTLESNEPFSLLPVLYDFKAELLVGERDFNNASP
YYEKAVQLWEKWLHPQVSLAKVYLEQGEFTSAAGVLKNVISKNAKHKEAKILLGIIEYRGFKKTDSAYSY
LSTALESKGRLSQLVEADGYAILAEIYVLRGEKSRALDAAQKGFSLNPNNNEIRQLVVRLGGSDKIKGEK
GQNNELLYLGDQYVRQGDFLAAQAEFKAAFEVDPKNGTAAMKAAKCLWQLNQSFEAIEWLNKAIKAEPKL
VSAYVLQADYMSQRFDFVGALQVLTNATRIAPNNYEVLRGLAMLEFRKNNMVGTINYAMRAAKVYDGDIE
TFILLSKANGLLARSIMPINKKEIERKENAAKDAIRYATKAVEIDATNPEAQITYAKMLAQTNGVDSGIN
YLNELIKRFSYTLDYRIALAEVYKSEDRYSQAKEIYEKVVEADPKSKKGWLGLGESEKALGLNDKALKAF
LSAAVLDPTDGEALFQAGKLYLETSRFEEAIQQFKRVQRLNGNYPRTYYYIGKAAFSSGDFATALEASKS
EKKLNPNVADSYILAAEVYTARKQYAECAAEYSHAMKLRPQGADIYVKSATCYRQSGSLDVAEDMLALAA
ARESGFAEIYREQGAIYELKGDTRSAAQAYNKYLGLSPNALDRAEIENKILRLGN

>B.spNC01
MSRIQVTWVVKTRTGQVKGPYSTEAILRMIGEGVFSGQEMISKLPDGQWTLISKEPAFYDKLLEALEGVV
EVDPKKAQKMEAETVIMRPPPTQQPKQEAPKPLNFENIAPPANSKLPTVDIYQQPAQIAPASIPVPNISS
NEGKDKVIELSNLKNMEKGELFKALRGPAAVLVVVILVAVFLLWDTGPAKGDKIHLLAPGKAGASLSETQ
VKDKLNEALFSIEQDTFDSYLDAQNKLVGIIEGAPSNIEVRALLCVVYRELWPYAKQDAQDVKTITMVTQ
ATRALNVVSPLGQVCESVKLMTSGRYREARGTVEATLESTEPFSLLPVLYGYKAELLQGEKDYLNAVPYY
EKSIQLWDKWLHPQVQLGVLYMEQQSFADASRMFQEVLKKNPRHREAKFLLGVVEYRGYKKSDSAFTLLS
SAADSKMRVPSLIEAQGLHALAEIYVLRNEKRKALETAQKAFSLNPNNSDLRQLVVRLGGSDKVKADKNQ
NNEMLFLGDQYVRQGDNLAAQAEFKAAFEADPKNGTAAMKAAKALWQLNQSFEAIEWLSKAMRAEPKLIS
AYVLQADYMSQRFDFNGAMMALTNATRISPNNYEVQRGLALLEFRKNNMIGAISYGTRAAKIYDGDIETF
ILLSKANAILAQSIVPLNKKEIERKENAGKDAVRYATKAVEIDATNPEAQITYAKTLAATQGVDAGITYL
NELIKRFSYTLDYRVALAEVYKSEDRYTQARDIYEKVVEADPRNKKAWLGLGEGSKALGMNDKALKAFLQ
AAVLDPTDGEALFRAGQLYLETSRFDEAIQQFRRVQRLNPNYPRTNYYIGKAAFASGDFNGAMEAAKAEK
RLNPNVADSYILAAEIYTARRQYQECAGEYSQAMKLRPQGADIYVKSAQCYRQSGAIDVAEDMLALAAAR
ESGYADIYREQGAIYEMKGDTRSAATAYNKYIGLSPNAPDRAEIEARINRLGN

>B.b_W
MGQVKGPYSTDAILRMIGEGIFSGQEMISKLPDGQWTQISREPAFYDKLLEALEGVVEVDPKKAQKMEAE
TVISSMPRPNSQAKPSASQENTGPITPNQLANLKIEKPKHQVDVFDRPQEIAAPTLPKRQSPQGGGNDGV
IELSNLKETESDDLKKRLRLPLVFIALAMILVVAFFWDSSPSSGDKQHLLAPGPSSGTLSDTQVKQKLNE
ALLAMEQDTYESYVSAQNKLVSIIEGAPMNIEVRGLLCVVYKQLWPFAVQDAQDIRTIAQVTQGARAINV
VSPFGQVCESVKLLTAGRYKEAKGVVEATLESNESFSLLPVMYAFKAELLEGERDFLNAVPYFEKSIQFW
DKWLRPQVLLGRLLLDRGEYLESSKYLKNVLQKNSKHREAKVLLGILEYRGFKKSDTAYTYLSSAMTEKA
KINQLTEADGLYVLSEILLLRSEKAKALEAAQRGYQLNPNNSELRQMVIRLGGSDKVKGEAGKNNELLYL

GDQYVRQGDCLAAQAEFKAAFEVDPKNGTAAMKAAKCLWQLNQSFEAIEWLNKAMKAEPKLVSAYVLQAD
YMSKRFDFVGATKVLTNAMRVSPNNYEILRGLALLEFRKNNMVGAINYASRSVKSYDGDIETYILLSKAN
GILARSILPVNKKEIDRKEAAMKDAIRFATKAVEIDATNPEAQVNYAKMLAQTNGAESGINYLNELIKRF
SYTLDYKIALAEIYKLEDRYNQSRDVYEKVVELDPRSKVAWLGLGESEKALGLNDKALKAFLSAAVIDPS
DAEALFQAGLLYLETSRFDEAIQQFKRVQRLNPNYPRTYYYIGKAAFSSGDFTTALEASKAEKKINPNVA
DSYILAAEVFTARRQYAECAGEYSHAMKLRPQGADIYVKSATCYRLSGSLDVAEDMLTLAAARESGYAEI
YREQGAIYEAKGDLRSAGTAYSKYLGLSPNAPDRAEVESKLSRLGM

>B.b_SKB
MSRIQVTWVVRTRSGQVKGPYSTEAILRMIGEGVFSGSEMISKLPDGQWSQISKEPAFYDKLLEALEGVV
DVDPKKVQKMEAETVIMKPPTSSGGTNSTIPNAPSRESLANIKVDPLAGRKVDIYDSPAHVTPASIPGSV
TSNTATIKTGGNSKVIDLSNLRTMEKDQLVKTLKVPLVGLAVIVLLGIWLLWDTGPARGDKIHLLAPGPN
SAPMSDGDVKKKMNEALFAMEQDTFESYLDAQNKLVSIVEGSSSNLEVRGLLCVVYRELWPFAKQDAQDQ
KTIAAVTQGTRAINVVSPFGRVCEAVKLLTMGRYREARGNIEATLESSEPFSLIPILYAYKAEMLELEKD
YNNAVPYFEKASQLWEKWLHPQVLLGKLYMSLGKYNEASQIFRTVLTRNPRHREAKILMGITEYRGFKKA
DSAFGFLNAAMESKSLVPPLTESSGLQVLAEIYVERNEKKKALDSAQKAFALNPNNGDLRQLVLRLGGSD
KVGGGKAQNNELLFIGDQYVRQGDYLSAQAEFKAAFEADPKNGTAALKAAKALWQLNQSFEAIEMLNKAI
KAEPKLISAYVTQADYLSQRFDFIRATTVLTSAIRISPNNYEVLRGLALLEFRKNNMAGAVNYGMRALKA
YDGDIDTYILLAKANGALALSIMPLNKKEIERKEAAGKDSVRYATKAVEIDGTNPDAQITYAKMLASTNG
VDAGVTYLNELIKRFSYTLDYRVALAEVMKSEDRWSQAKDIYEKVVEADQKNKKGWLGLGESYKALGLND
RSLKAFLQAAVLDPTDGEALFQAGKLYLETARYDEAINQFKRVQRLNPNYPRTWYYIGKAAFASGDLNTA
VDAAKQEKKLNPNLAEPYILAAEVYAAKKQFTECAGEYSQAMKLRPQGADIYVKAAQCYRQSASIEVAED
MLALAAARESGYAEIYREQGAIYEIKGDIRSAAQSYNKYLGLSPNAPDRAEIENKLNRMGN

>B.spZAP7
MSRIQITWVVRTRSGQVKGPYSTEAILRMIGEGVFSGQEMISKLPDGQWTQISKEAAFYDKLLEALEGVV
DVDPKKVQKMEAETVIMKPPSGQTNSSSTIPGGPTKDSLANIKVDPNAGRKIDIYDSPSHVAPVTIPGTI
SSNKTKTDAKATVIDLSNLKNMEKDQLVKTLKLPLLGLAAVILIGIWLLMDDGPARGDKIHLLAPAKQTS
PMSDADVKKKLNEALVAMEYDTFESYLDAQNKLVSIVEGSSSNLEVRGLLCVVYRELWPFAKQDAQDFKT
ISAVTQSTRAINVVSPFGRVCEAVKLLTLGKYREARSNIEATLESPEQFSLVPILYTYKAELLELEKDYN
NAVPYFEKASQMWDKWLHPQVMLGKLYMSVDKYNEASQVLRNVLAKNPKHREAKILMGITEYRGFKKSDS
AFSFLSAALESKSQVSPIVESTGLQVLAEIYVERNEKKKALSMAQKAFALNPNNGGLRQLVLRLGGSDKI
SGGKNQTSDILFLGDQYVRQGDYLAAQAEFKAAFESDPKNGTAALKAAKALWQLNQSFEAIEWLNKAIKA
EPKLVSAYVTQADYLSQRFDFAKATTVLTNAIRIAPNNYEVLRGLALLEFRKNNMPGAVNYGMRSLKAYD

GDIETYILLSKANGALALSIMPLNKKEIERKEIAGKDAVRFATKAVEVDGTNPEAQITYAKMLAATNGVD
SGITYLNELIKRFSYTLDYRIALAEVMKSEDRWNQAKDIYERVVEADQRNKKGWLGLGESYKALGLNDKS
LKAFLQAAILDPSDGEALFQAGKLYLETARYDDAIKQFKRVQATNPNYPRTWYNIGKAAFSSGDLNGAVE
AAKMEKKLNPNIADSYILAAEVYAARKQFTECAGEYSQAMKLRPQGADIYVRAAQCYRQSASLEVAEDML
ALAAARESGYAEIYREQGAIYEIKGDIRSAAQAYNKYLGLSPNAPDRAEIENKLNRLGN

>B.spKM01
MSRIQITWVVRTRSGQVKGPYSTEAILRMIGEGVFSGQEMISKLPDGQWTQISKEPAFYDKLLEALEGVV
DVDPKKVQKMEAETVIMKPPSGQTNSSSTIPGGPTKESLANIKVDPNAGRKIDIYDVPSQVAPVTIPGTI
SSNKTKTDAKATVIDLSNLKNMEKDQLVKTLKLPLLGLAAVILIGIWLLMDDGPARGDKIHLLAPAKQSS
PMSDADVKKKLNEALVAMEYDTFESYLDAQNKLVSIVEGSSSNLEVRGLLCVVYRELWPFAKQDAQDFKT
ISAVTQSTRAINVVSPFGRVCEAVKLLTLGKFREARSNIEATLESPEQFSLVPILYTYKAELLELEKDYN
NAVPYFEKASQMWDKWLHPQVMLGKLYMSVDKYNEASQVLRNVLAKNPKHREAKILMGITEYRGFKKSDS
AFSFLSAALESKSQVSPIVESTGLQVLAEIYVERNEKKKALSMAQKAFALNPNNGGLRQLVLRLGGSDKI
SGGKNQTSDILFLGDQYVRQGDYLAAQAEFKAAFESDPKNGTAALKAAKALWQLNQSFEAIEWLNKAIKA
EPKLVSAYVTQADYLSQRFDFAKATTVLTNAIRIAPNNYEVLRGLALLEFRKNNMAGAVNYGMRSLKAYD
GDIETYILLSKANGSLALSIMPLNKKEIERKEIAGKDAVRFATKAVEVDGTNPEAQITYAKMLAATNGVD
SGITYLNELIKRFSYTLDYRIALAEVMKSEDRWNQAKDIYERVVEADQRNKKGWLGLGESYKALGLNDKS
LKAFLQAAILDPSDGEALFQAGKLYLETARYDDAIKQFKRVQATNPNYPRTWYNIGKAAFSSGDLNGAVE
AAKMEKKLNPNIADSYILAAEVYAARKQFTECAGEYSQAMKLRPQGADIYVRAAQCYRQSASLEVAEDML
ALAAARESGYAEIYREQGAIYEIKGDIRSAAQAYNKYLGLSPNAPDRAEIENKLNRLGN

>B.Exovorus
MSDNSGGQWVIKIQSDQVKGPYSTDAVIKMILQGVFSGNEEICAYPEGEWKALTKQPEFYDALLESLENP
VEVDNKKTQKMEAETVVKAVEVAPVAADEMPPIPELKTSDDLKEFLEKELNKDKDEENKPEKSRRNRSLA
RTELMPQVQSIPVNPGAEMIANRDQNLEIQMSDLEDLKQKEMGKLLPFILLCIVAVCAVIYLLWPENQTE
KKGWALYAPKKGAEALEETEVRNLKVKAVRALQSGIYEQILVAQQDMVRAVEGAPKDLEAMGLLCMIYEQ
LWPYTRQTIEGDIRSVMVVTQMARTLNAISNYSNSCQAVYLSVLGRGREARSLVEKTLDNQTEEKFSLGP
FLYLMKAQMLEAEGTTVNAAAYYEQAMKLWPQWMIARFGLARMLFKQNKYEEARTQYEQMYEFNKESKAA
LFGLGLVENKGLRNPEKAYTFYTNGFRLKQVLPKDFATEALQNYAQLLMEKNENKKALEAAQEGYRLSPS
HRGLKEMVVSLGGDEKVENAQSEIMLIGDQFFRNGDYMVAQAQYKTAFELDTKNGLAAYKAARALWLMNQ
TRDAILWLDKSIEADPKLLPAYVLKSDYESQKYNFLEAAKTLQKASRAFPQNHEVLKAQALLEFRKNNMM
GAIQYGERAVKLYSADVELLTLLAQAHIYFYVNAPATRQQDLDRKEASKTAAQRYAGRAVDLEPSWPESQ
ITWAKVLSATDGPTRGQNYLKEMIKAFPYTLDYRIALAEFYRDSEKFLDSSKVYEEVVSIDPKSKRASLG
LAEAYRILNKPDLAQKYYNITSVLDPSDVEPMVANARLLVETAAGNEVRAKMQQALTKLLLVKKINPDFP

KVSFLMAKCYMELGDYDKAIEMIKEEKTRNPNIADSYILAAEIFFRRQQYKECAVEYSAATRMRPSSAEL
YVRASTCYRMSDSIDIAEDMLNIAAQKESGFADIYRELGYIYERKNGGRVQAVQYFRRYLSLSPNAPDRT
TVEGRIRQMGEQP

>B.sp_qaytius
MGKRVNFNDQWRIKMENGQIKGPFPSESISKMIVEGILSGQEEVAQHPSAEWRPLSKVHEFYEALLESLE
NPTERDEKKALKMDAETQIQAQSTPKKAPLKEEADDVEPKPIPEFKDDIKQFVQDEIKKASNAPALVPGQ
TPRSPQLPVAAPGAETDALLKARDEQLTIEMQQLKTIQARESKKFIPFVAIILAVIVGFYFLLSAGDKKS
NGWVLIAPNRGAEKKLTENEVRILKTRAITLIKSGTLENTRDSQKFLAQASENAPNDLEVLGLLCVAYEQ
LWPYTKQNSNDLKAITMVTQMSRTINPISNYSDTCQSVYLLAKGQPRDARGLIEKTLDQQAGERFILFPF
LYLMKGQILEEMQNHISAEAYYVEAVKSFPGWAWAEFALARSYYKQGKINEAKDAYTNILRDYPEYKGAL
YGMGLVELKTSGTDKALSYFTRAYAIDGKLPKSFHMDALQEYMQLLISKKDSSKALDVGQFALTISPSHR
AIKELVMSLGGEDRGNNQSAELVFLGDQFARSGDHLAAQAQYKAAFEFDKSNAEPALKAAKSLWAINQSR
EALTWVDRAIKADPKLSQAYAQKADYLSQKYAFGEASKTLQDAMRAGGLNFDVVKTQALVEYRKNSMNTA
ITYGEKAIKMYDADTELLSLIANANINLFLNSPSRTKEEENRKAKYLEDAQKYSSKAVDLEPGKPEAQIT
YSKYLYAKQGNIAAETNFKKLIETFPYTIDYRLGLADFYELQEKYRSASEIYAQIVETDPKNKKALMGLA
HSNQFMNEIKIAQKYYMDAAVLDPSDVEPLFATAQLELENPGSKDASAVIQSAYKKFEMVKRVNPSYPRI
SYFLARCHFEMGEFDKALEMIKDEKTKNPGIADPYLLAATVYNAKEQYKECAAEYSQGIRLRPTSADLYV
KAANCYRKSDAFDIAADMLDIARQKESGFAPIYKEKAYIMEAQGDRKSARELFQLYIELSPNALDRKQIE
DKISSLRGN
