# Supplementary material for: Using AlphaFold-Multimer to study novel protein-protein interactions of predation essential hypothetical proteins in Bdellovibrio
Source: Front Bioinform. 2025 Apr 14;5:1566486. doi: 10.3389/fbinf.2025.1566486 (PMC12034629; doi:10.3389/fbinf.2025.1566486)
Supplement: Supplementary file 4 [file DataSheet4.pdf]

Supplementary 5

3D homology search using Foldseek

CATH50 (Class, Architecture, Topology, and Homologous superfamily Database)

TM-Score: 0.78372
RMSD: 1.37

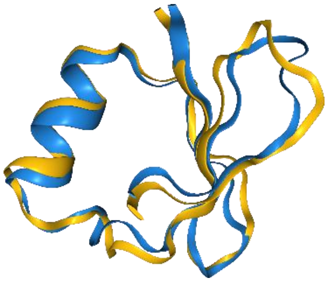

UniProt: O36025 381-458
GYF domain
(*Schizosaccharomyces pombe*)

TM-Score: 0.71837
RMSD: 2.68

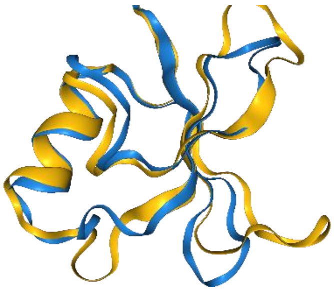

UniProt: O95400
GYF domain
(*Homo sapiens*)

TM-Score: 0.68333
RMSD: 3.58

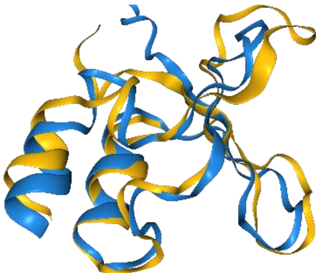

UniProt: Q0D8Q3
GYF domain
Oryza sativa

AFDB50 (AlphaFold Protein Structure Database)

TM-Score: 0.88263
RMSD: 1.13

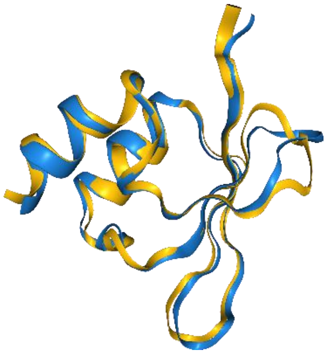

UniProt: A0A382EVS8
GYF_2 domain-containing protein
(marine metagenome)

TM-Score: 0.83602
RMSD: 1.91

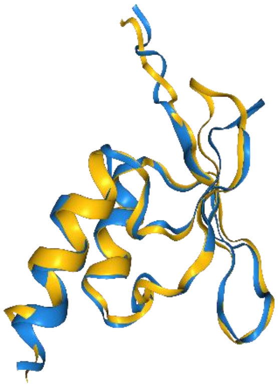

UniProt: A0A383AJS8
Cyclic nucleotide-binding domain
(marine metagenome)

TM-Score: 0.84311
RMSD: 2.73

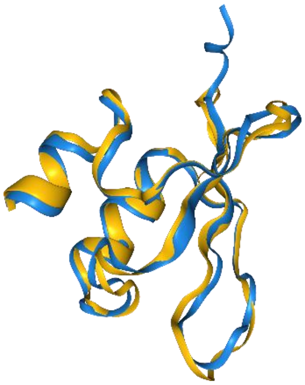

UniProt: A0A2N2IH23
FHA domain containing protein
(*Deltaproteobacteria*)

AFDB-PROTEOME (AlphaFold Database-PROTEOME)

TM-Score: 0.71138
RMSD: 2.43

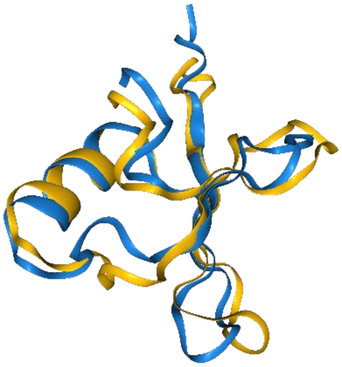

UniProt: Q9NAH3
Tumorous Enhancer of Glp-1(Gf)
(*Caenorhabditis elegans*)

TM-Score: 0.72559
RMSD: 2.67

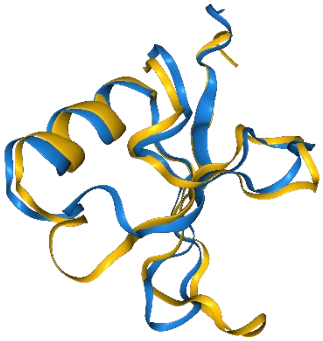

UniProt: A0A044V700
GYF domain-containing protein
(Onchocerca volvulus*)*

TM-Score: 0.63097
RMSD: 11.94

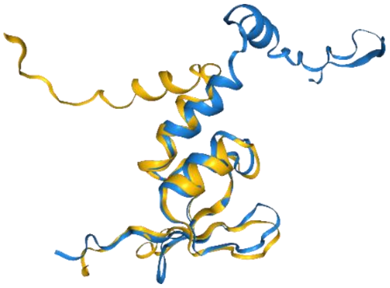

UniProt: C0HP9
RNA-binding protein, putative
(*Plasmodium falciparum* 3D7)

BFVD (Big Fantastic Virus Database)

TM-Score: 0.72467
RMSD: 2.66

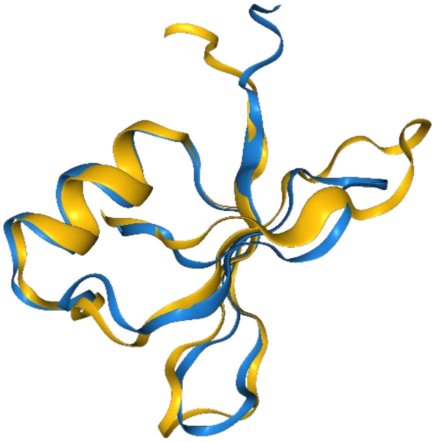

UniProt: Q4A2R6
Uncharacterized protein
(*Coccolithovirus*)

TM-Score: 0.61346
RMSD: 3.11

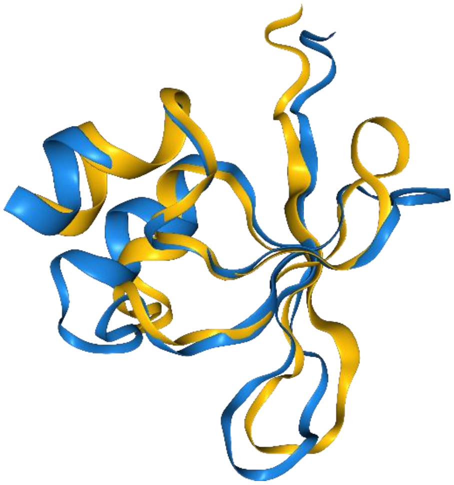

UniProt: U5XK58
Uncharacterized protein
(*Phormidium* phage MIS-PhV1B)

TM-Score: 0.4673
RMSD: 16.77

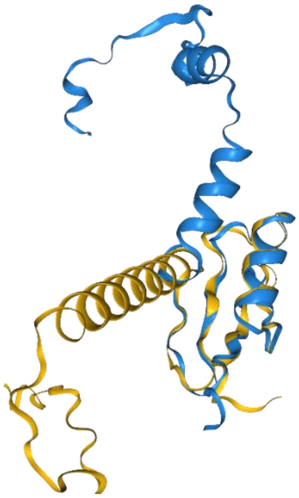

UniProt: A0A8S5T7N5
GYF domain 2
(*Myoviridae sp*. cta6i12)

Figure. Showing 3D homology hits with their respective TM and RMSD scores from four databases within Foldseek. Querry protein, Bd0075A-domain is shown in blue while corresponding portions of the hit proteins are shown in yellow. UniProt accession numbers and the name of the organisms where the hit proteins can be found are provided respectively.

Structural similarity of the Bd2209-B22212 complex to the Bd2723-Bd2726 complex

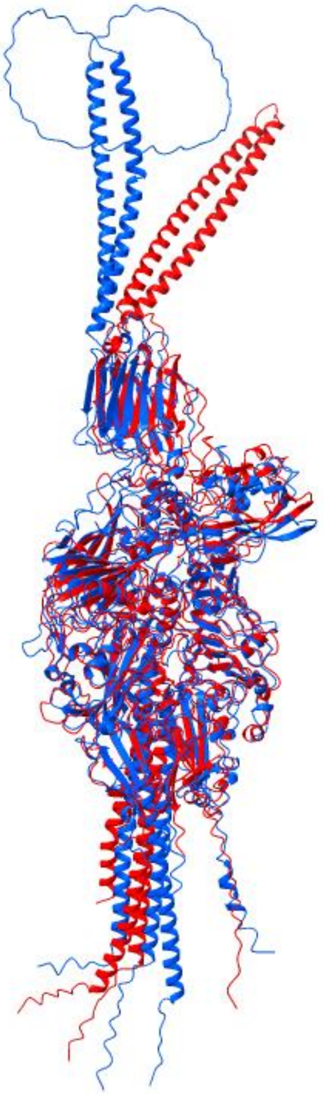

Aligned length= 1634, RMSD= 4.17 Å,

TM-score= 0.80356 (normalized by length of Bd2209-Bd2212 complex: Length=1890, d0=13.49)

TM-score= 0.85455 (normalized by length of Bd2723-Bd2726: Length=1772, d0=13.16)

Figure. Superimposed structure of Bd2209-Bd2212 complex (Blue) and Bd2723-Bd2726 complex (Red). TM-score =0.80356, RMSD = 4.17 Å

AlphaFold multimer model of the Interaction between Bd0075 and Bd0474

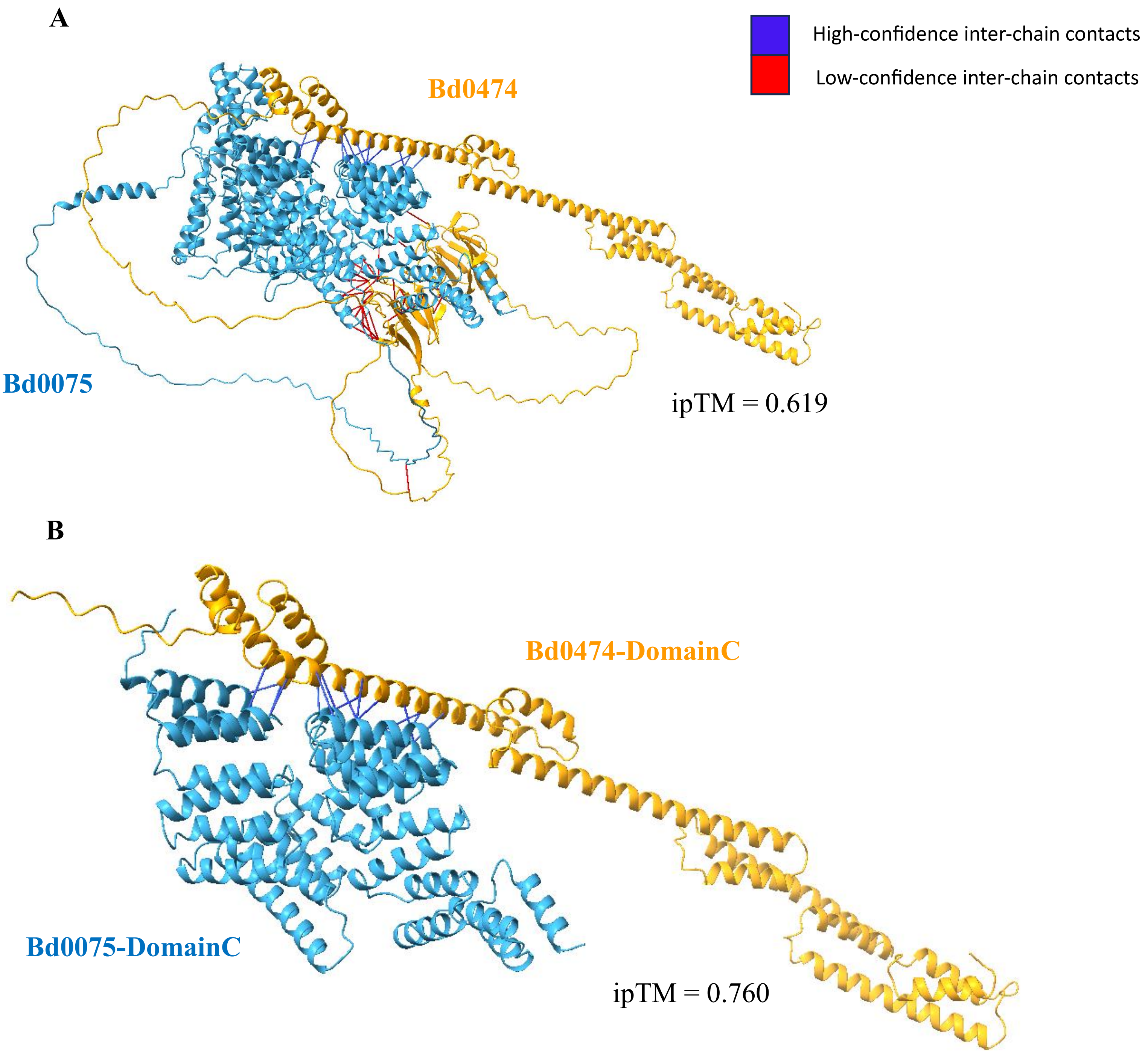

Figure. (A) Interaction model of Bd0075 and Bd0474. Blue lines indicate high-confident interactions while red lines indicate low-confidence (uncertain and unfavourable) interactions. Note that red lines in this model correspond to bonds between domains on different sides of the membrane and would be spatially unavailable for interaction. (B) Model showing Bd0075-DomainC and Bd0474-DomainC only.
